# Targeted degradation of oncogenic BCR-ABL by silencing the gene of NEDD8 E3 ligase RAPSYN

**DOI:** 10.1186/s12951-024-02505-5

**Published:** 2024-05-13

**Authors:** Yanzi Sun, Yishu Wang, Chunyan Liu, Yingshuang Huang, Qiulin Long, Caoyun Ju, Can Zhang, Yijun Chen

**Affiliations:** 1 State Key Laboratory of Natural Medicines and Laboratory of Chemical Biology, China Pharmaceutical University, 639 Longmian Ave, Nanjing, 211198 Jiangsu China; 2 State Key Laboratory of Natural Medicines, Jiangsu Key Laboratory of Drug Discovery for Metabolic Diseases, Center of Advanced Pharmaceuticals and Biomaterials, China Pharmaceutical University, Nanjing, 210009 People’s Republic of China

**Keywords:** Ph^+^ leukemia, RAPSYN, Lipid nanoparticles, siRNA, CD79B, scFv

## Abstract

Tyrosine kinase inhibitors have been the standard treatment for patients with Philadelphia chromosome-positive (Ph^+^) leukemia. However, a series of issues, including drug resistance, relapse and intolerance, are still an unmet medical need. Here, we report the targeted siRNA-based lipid nanoparticles in Ph^+^ leukemic cell lines for gene therapy of Ph^+^ leukemia, which specifically targets a recently identified NEDD8 E3 ligase RAPSYN in Ph^+^ leukemic cells to disrupt the neddylation of oncogenic BCR-ABL. To achieve the specificity for Ph^+^ leukemia therapy, a single-chain fragment variable region (scFv) of anti-CD79B monoclonal antibody was covalently conjugated on the surface of OA2-si*RAPSYN* lipid nanoparticles to generate the targeted lipid nanoparticles (scFv-OA2-si*RAPSYN*). Through effectively silencing *RAPSYN* gene in leukemic cell lines by the nanoparticles, BCR-ABL was remarkably degraded accompanied by the inhibition of proliferation and the promotion of apoptosis. The specific targeting, therapeutic effects and systemic safety were further evaluated and demonstrated in cell line-derived mouse models. The present study has not only addressed the clinical need of Ph^+^ leukemia, but also enabled gene therapy against a less druggable target.

**Graphical Abstract:**

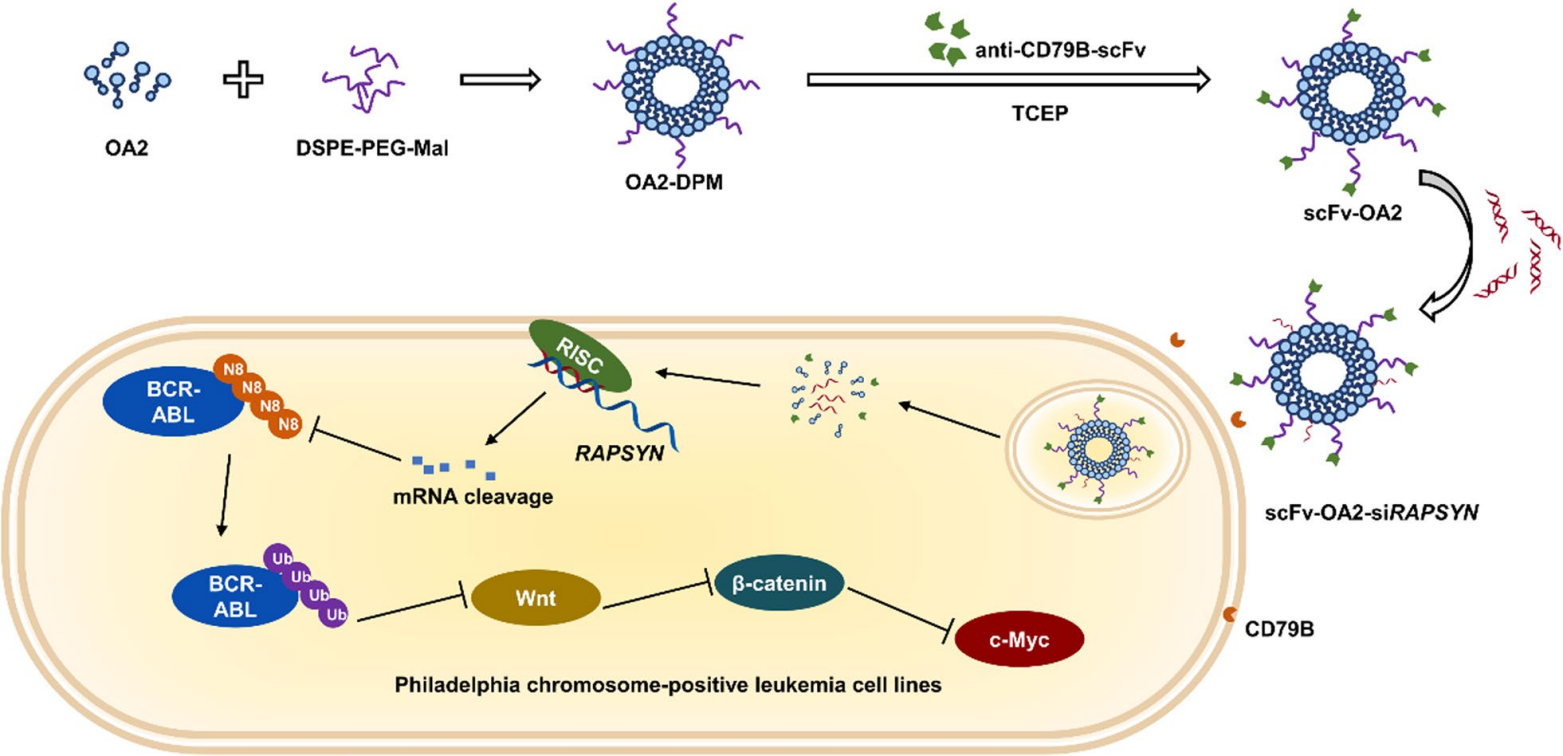

**Supplementary Information:**

The online version contains supplementary material available at 10.1186/s12951-024-02505-5.

## Introduction

Philadelphia chromosome positive (Ph^+^) leukemia is a malignant clonal disease derived from hematopoietic stem cells, which is characterized by the expression of BCR-ABL fusion protein possessing strong tyrosine kinase activity [1, 2]. Although tyrosine kinase inhibitors (TKI) are the only approved treatment option for Ph^+^ leukemia, the nature of resistance, intolerance and recurrence has forced to evolve multiple generations of TKI, from imatinib to panatinib [3, 4]. Meanwhile, according to clinical data, first-line use of TKI is unable to bring huge clinical benefits to patients based on risk–benefit assessments [5, 6]. Therefore, merely inhibiting the kinase activity of BCR-ABL is not an ultimate solution for Ph^+^ leukemia patients, which remains as an unmet medical need [7].

Given the definitive relationship between oncogenic BCR-ABL and the occurrence of Ph^+^ leukemia, effective degradation of this fusion protein, rather than the inhibition of tyrosine kinase activity, would be a favorable choice to overcome a series of drawbacks of small molecule inhibitors. Recently, we found that RAPSYN, a scaffolding protein possessing NEDD8 E3 ligase activity, can neddylate BCR-ABL to compete c-CBL-mediated proteasomal degradation, and therefore silencing *RAPSYN* gene could effectively inhibit the progression of Ph^+^ leukemia [8]. In addition, the disruption of neddylation could not only induce apoptosis of the leukemic cells with BCR-ABL mutations [9], but also eradicate leukemia stem cells (LSCs) [10], strongly suggesting the advantageous outcomes of targeting this neddylation processes. Thus, these studies have laid a solid foundation for RAPSYN as a potential therapeutic target for Ph^+^ leukemia.

As a scaffolding protein, RAPSYN has been reported to extensively express in 87 tissues of a human body, with the highest expression in leg muscles [11]. If RAPSYN was pathologically absent, severe diseases, such as myasthenia gravis, would occur [12–14]. All these facts make RAPSYN as a less druggable target. To transform this widespread protein to a druggable target, targeting *RAPSYN* gene in Ph^+^ leukemia cell lines with high gene specificity and cell selectivity is absolutely required to ensure the safety of the treatment. Therefore, how to maintain a sufficient period for the therapeutic agents in blood circulation and how to specifically target Ph^+^ leukemic cells are a challenging task for the treatment of Ph^+^ leukemia by the approach of gene therapy.

As commonly used for antigen targeting, single-chain fragment variable (scFv) derived from conventional antibodies, consists of a variable domain from the heavy chain and another variable domain from the light chain [15]. Because of the lack of Fc fragment, scFv does not exhibit ADCC, ADCP and CDC effects in addition not to activate the immune system. Moreover, given their smaller size, scFv portions usually possess better cellular penetration capability than conventional antibodies and easier linkage with various vehicles as warheads for the binding with specific antigens, which could greatly facilitate targeted therapy [16]. According to a previous report [17], CD79B is highly expressed on the surface of acute lymphoblastic leukemia cells. Thus, it is highly possible to utilize CD79B as an antigen for targeting Ph^+^ leukemia by its corresponding scFv.

In this study, we fabricated a *RAPSYN* and CD79B dual targeted lipid nanoparticle (scFv-OA2-si*RAPSYN*) via covalent conjugation of anti-CD79B-scFv on the surface of the lipid nanoparticles (OA2-si*RAPSYN*) composed of single cationic lipoid OA2-based liposomes and *RAPSYN*-targeted siRNA. This dual targeted lipid nanoparticle could effectively promote the degradation of BCR-ABL. Consequently, the present gene therapy may represent a new treatment approach for hematological tumorigeneses.

## Materials and methods

### siRNA synthesis

si*RAPSYN*, NC-siRNA was synthesized by RiboBio Co., Ltd. (Guangzhou, China). FAM-labeled siRNA (Fam-siRNA) and Cy5-labeled siRNA (Cy5-siRNA) were prepared by Sangon (Nanjing, China).

### Cell cultures

The cell lines of K562 (female; CBP60529), MEG-01 (male; BP61104), KU812 (male; BP60732) were purchased from COBIOER in a mixture containing 10%-20% fetal bovine serum (FBS; FS301-02, TransGen Biotech) and 100 mg/mL streptomycin/penicillin (FG101-01, TransGen Biotech). HS-5 cells (male; CRL11882) were from American Type Culture Collection (ATCC) in Dulbecco’s modified Eagle’s culture-medium (1640; KGM12800, KeyGEN BioTECH) containing 10% FBS and 100 mg/mL streptomycin/penicillin. All cells were cultured in a moisture incubator containing 5% CO_2_ at 37 °C. All cell lines were identified by short tandem repeat matching analysis and were free from mycoplasma contamination.

### Animals

NOD/ShiLtJGpt Prkdcem26Cd52Il2rgem26Cd22/Gpt (NCG) female mice (4–6 weeks) were from GemPharmatech Co., Ltd (Nanjing, China). Mice were placed in a barrier facility under pathogen-free conditions of 24 ± 1% and 55 ± 5% humidity for a light–dark cycle of 12 h. All animal experiments were conducted in compliance with the WMA Statement on animal use in biomedical research, and approved (No. 2020-07-009) by the Animal Care and Use Committee of China Pharmaceutical University (Nanjing, China).

### Cell transfection of the liposomes

Transfection of the indicated siRNA into cells was performed with the liposomes prepared with OA2, TA7 and TA13 as well as commercial riboFECT™ CP (Ribobio, Guangzhou, China). Briefly, when the cell confluency reached 50%, siRNA and liposomes were diluted in deionized water and incubated for 30 min at room temperature, respectively. Then, the mixtures were added into the target cells. Transfected cells were collected after 48 h for further analyses.

### Gene knockdown in vitro

K562, KU812 and MEG-01 cells were inoculated into 6-well plates at a density of 1 × 10^6 cells per mL. Cells were incubated with OA2-siNC or OA2-si*RAPSYN* at a dose of 1 μg/mL siRNA in a humid atmosphere of 5% CO_2_ at 37 ℃ for 48 h. According to the manufacturer’s instructions, total RNA was extracted using TRIzol® reagents (Invitrogen, CA, USA), and the RNA was reverse-transcribed to cDNA using Evo M-MLV reverse transcription premix kit (Accurate Biology, Nanjing). Then, SYBR Green Pro Taq HS premixed qPCR kit (Accurate Biology, Nanjing) was used to detect the transcriptional levels of target genes in each group. A two-step RT-PCR was performed on a Biosystems StepOnePlus real-time fluorescence quantitative PCR apparatus. Sample analysis was conducted in triplicate. The level of target gene in a given sample was normalized to the corresponding level of GAPDH. The relative transcription of target gene was calculated by 2^-∆∆Ct^ method.

The primers used in qRT-PCR for *RAPSYN* and GAPDH were as follows:

- 5’-GTACGACTCCGCCATGAGCA-3’ (*RAPSYN* Forward primer);
- 5’-TGGCATCCAGAGCCTTGTCC-3’ (*RAPSYN* Reverse primer);
- 5’-CTCTGATTTGGTCGTATTGGG-3’ (GAPDH Forward primer);
- 5’-TGGAAGATGGTGATGGGATT-3’ (GAPDH Reverse primer).

### Western blot

Total proteins were obtained by cell lysis (Beyotime, Nanjing, China), and protein samples of the same concentration were electrophorized on 8% or 10% SDS-PAGE and then transferred to PVDF membranes (Millipore, USA). After incubating with anti-RAPSYN (Abcam, Cambridge, GB), anti-BCR-ABL (Abcam, Cambridge, GB), anti-Wnt5b (Abcam, Cambridge, GB), anti-c-Myc (Proteintech, Wuhan, China), anti-β-catenin (Proteintech, Wuhan, China), and anti-β-Tubulin (Proteintech, Wuhan, China) overnight at 4 ℃, then incubated with secondary antibody of goat anti-rabbit IgG/HRP (CST technology, Boston, USA) or goat anti-mouse IgG/HRP (CST technology, Boston, USA) at room temperature for 2 h. Finally, protein bands were visualized using ECL system (Tanon 3500).

### Cell viability assay

The cells were inoculated into 96-well plates with a density of 10,000 cells per well, and incubated with 1 μg/mL siRNA or siNC for 24, 48, 72, 96 h. The cell viability was measured using CCK-8 assay kit (Vazyme, Nanjing, China) according to the manufacturer’s instructions. Each experiment for each cell line was repeated for at least three times.

### Cell apoptosis assay

The apoptosis index of cells treated with OA2-siNC or OA2-si*RAPSYN* for 24 h was detected by annexin V-FITC Apoptosis Test kit (BD Biosciences, San Jose, USA). Flow cytometry was employed to analyze the cells with the software of FlowJo version 10.6.1.

### Cell proliferation assay

According to the protocol of the CFDA SE cell proliferation and tracer detection kit (Beyotime, Nanjing, China), cells with logarithmic growth stage were labeled, and then OA2-siNC or OA2-si*RAPSYN* was added, respectively. Cell division was monitored by measuring CFSE using flow cytometry via channel 488 at day 0, day 2, and day 4.

### Determination of the neddylation of BCR-ABL

One mg of total protein extracted from cells was incubated with IgG or BCR-ABL antibody for 1 h, and then Protein A/G agarose strain (Bioworld, Nanjing, China) was added for overnight incubation at 4 ℃. On the second day, after washing the beads with PBS for 4 times, sample buffer was added and boiled for protein denaturation. Protein concentration of the cells was adjusted to the same for sampling, and separated by 6% SDS-PAGE for the transfer to PVDF membrane. Then, 5% skim milk powder was added and incubated with anti-BCR-ABL antibody (Abcam, Cambridge, GB) or anti-NEDD8 antibody (Abcam, Cambridge, GB) at 4 ℃ overnight. Subsequently, the samples were incubated with corresponding secondary antibody at room temperature for 2 h. The target bands were visualized using ECL system (Tanon 3500).

### Detection of CD79B expression

A 24-well plate was coated with Poly-D-Lysine (Beyotime, Shanghai, China), and then 2 × 10^5 K562 cells were inoculated into each well. After the attachment of the cells with plate wall, anti-CD79B antibody (Proteintech, Wuhan, China) was added to incubate at room temperature for 1 h. Then, the secondary antibody labeled with 488 dye (CST technology, Boston, USA) was added and incubated for 1 h. Finally, the conjugates were incubated with DAPI for 5 min for the comparison under fluorescence microscope.

### Expression and purification of anti-CD79B-scFv

Plasmid construction: The amino acid sequence of anti-CD79B (L/H) scFv was from US patent USA20200207852, and codon optimization was performed to make it suitable for the expression in *Escherichia coli*. The gene fragment of MBP tag was inserted into pET28a vector of its N-terminus by using Gibson assembly kit, and then the codon-optimized gene (Additional file 1: Table S1) of anti-CD79B-scFv with a 6 × His tag on its C-terminus was inserted into the pET28a plasmid containing MBP tag to result in the expression vector of pET28a-MBP-scFv.

Protein expression and purification: The pET28a-based plasmid of MBP-anti-CD79B-scFv-6xHis was transformed to *E. coli* BL21(DE3) cells (Sangon, Nanjing, China). A transformed colony was inoculated into LB containing kanamycin to culture overnight with shanking at 220 rpm at 37  ℃. On the second day, bacterial culture (1:1000) was inoculated into LB medium containing 1 mg/L kanamycin and continued to grow under the same conditions. When OD_600_ of the culture reached approximately 0.8, 0.1 mM IPTG was added to the culture to induce protein expression at 18 ℃ and 200 rpm for 18 h. Then, the bacterial cells were collected by centrifugation at 8000*g* for 10 min, and mixed with 20 mL buffer (50 mM K_2_HPO_4_, 500 mM NaCl, 10 mM imidazole pH 7.5). After disrupting the cells by ultrasound at 120 w for 15 min, the supernatant was obtained by centrifugation at 12,000 × *g* for 30 min. Two affinity chromatographic steps were employed for the purification of MBP-anti-CD79B-scFv-6 × His. First, the soluble fraction was incubated with 300 μL Ni–NTA resin (GE HealthCare) at 4 °C for 1 h and then loaded onto a gravity flow column. The protein was preliminarily purified by the elution with the buffer (50 mM K_2_HPO_4_, 500 mM NaCl, 300 mM imidazole, pH 7.5). Next, the eluted protein was mixed with dextrin sepharose resin (Cytiva, shanghai, China) and loaded onto a column for another affinity chromatography, and the purification was achieved by the elution with the buffer (50 mM Tris, 500 mM NaCl, 10 mM maltose, 5% glycerol, pH 7.5). Finally, the purity of the recombinant MBP-anti-CD79B-scFv-6 × His was examined by 10% SDS-PAGE with Coomassie blue staining.

### Determination of binding affinity of scFv

The experiments were performed according to a previous report [18] with minor modifications. Different concentrations of anti-CD79B-scFv ranging from 0.0001 to 1 mg/mL were added to each well in 96-well plates and incubated at 37 °C for 2 h. Horseradish peroxidase labeled anti-His monoclonal antibody (Proteintech, Wuhan, China) was used as the secondary antibody.

### Preparation of the complexes of lipoids with si*RAPSYN*

TA7-si*RAPSYN* and TA13-si*RAPSYN* were prepared according to the procedures previously described [19].

### Preparation and characterization of OA2-si*RAPSYN* and scFv-OA2-si*RAPSYN*

For OA2-si*RAPSYN*, OA2 were dissolved in a mixture of 2 mL anhydrous methanol and 3 mL chloroform and transferred to a 500 mL solanum-shaped bottle. After rotary evaporation at 40  ℃ for 10 min, vacuum drying overnight was performed to thoroughly remove organic solvents. Then, an appropriate amount of deionized water was added to hydrate OA2 lipid film (final OA concentration of 2 mg/mL) at 37 ℃ for 30 min. The mixture was then extruded through 0.8 µm, 0.4 µm and 0.2 µm carbonate films for 11 times each to obtain the OA2 lipid nanoparticles. Finally, si*RAPSYN* was added into the solution of OA2 lipid nanoparticles at N/P of 5 at room temperature for 30 min to obtain the OA2-si*RAPSYN*.

Regarding scFv-OA2-si*RAPSYN*, OA2-DPM was first prepared with the same method of OA2 lipid nanoparticles except for adding the DPM at a ratio of OA2/DPM of 98:2 (mol/mol). Next, to obtain scFv with free sulfhydryl groups, tris(2-carboxyethyl) phosphine (TCEP) was dissolved in PBS at 2 µmol/mL and mixed with scFv at room temperature for 10 min, following the dialysis against PBS at 4 ℃ for 2 h to obtain the reduced scFv protein, which was then incubated with OA2-DPM at a ratio of DPM/scFv of 30:1 (mol/mol) at room temperature for 2 h to obtain scFv-OA2. The complex of scFv-OA2 and si*RAPSYN* were the same as mentioned before.

Particle size, polydispersity index (PDI) and zeta potential were determined by using Laser Light Scattering System (BL-200SM, Brookhaven, USA).

To determine siRNA stability, agarose gel electrophoresis was performed on 1% agarose gel at an electrophoresis condition of 140 V for 20 min and visualized by gel imaging system. OA2-si*RAPSYN* and scFv-OA2-si*RAPSYN* with or without 10% Triton X-100 solution were respectively sampled. Free siRNA was used as the control.

For TEM images, different samples were added onto the copper net and stained by 1% phosphotungstic acid. After drying, TEM was performed at 80 kV to observe the morphology (HT7700, Hitachi, Japan).

For in vitro stability, the particle sizes of OA2-si*RAPSYN* and scFv-OA2-si*RAPSYN* after incubation with saline or 1640 medium containing 10% fetal bovine serum (FBS) at 37  ℃ for different time points (0, 1, 2, 4, 6, 12 and 24 h) were respectively determined.

### Assays of protein concentration and coupling rate

To determine scFv coupling rate on liposome surface, BSA solution (0.5 mg/mL) of 0, 1, 4, 8, 12, 16 and 20 µL was respectively added to a 96-well plate. After addition of 20 µL ultra-pure water, each well was mixed evenly with 200 µL BCA reagent to establish a standard curve. Subsequently, freshly prepared scFv-OA2 liposome (500 µL) was centrifuged with a 100 kDa ultrafiltration tube at 5000 × *g* for 15 min to obtain the solution. The solution containing free scFv (20 µL) was added to each well in a 96-well plate. Then, 200 µL of BCA reagent was added, gently mixed and incubated at 37 ℃ for 30 min. The absorption at 562 nm was recorded with triplicated wells for each group. The concentration (Ci) of scFv in the solution was calculated according to the standard curve, and the coupling rate was calculated according to following formula:

$$Coupling \, rate \, = \, \left( {co - ci} \right)/Co \, \times \, 100\%$$

where, Co is the concentration of total scFv, Ci is the concentration of free scFv in the solution.

### Cellular uptake of scFv-OA2-si*RAPSYN*

K562, KU812 and MEG-01 cells were inoculated into 6-well plates at a density of 1 × 10^6 cells per mL. Cells were cultured with FAM-labeled siNC, OA2-siNC, scFv-OA2-siNC and scFv-OA2-siNC with scFv at a dose of 2 μg siRNA in a humid atmosphere of 5% CO_2_ at 37 °C for 12 h and then collected for flow cytometry analysis (BD FACS Celesta).

### The intracellular lysosomal escape of scFv-OA2-si*RAPSYN*

Poly-D-Lysine-coated cell slides were placed in a 24-well plate in advance, and K562 cells were inoculated at a density of 5 × 10^5 cells per mL. Then, cells were incubated with FAM-labeled siNC and scFv-OA2-siNC in a humid atmosphere of 5% CO_2_ at the dose of 1 μg siRNA for 6 h. The 24-well plates were taken out and washed twice with preheated hanks buffer (Vazyme, Nanjing, China). Then, Lysosomal stain (Abcam, Cambridge, GB) was added and incubated at 37 ℃ with 5% CO_2_ for 30 min, and then washed twice with hanks buffer at 37  ℃ and fixed with 4% paraformaldehyde for 15 min. The nuclei fractions were stained with DAPI for 5 min, and the slides were removed after three times of washing with PBS. A drop of fluorescence quencher was dropped on the slides, after fastening and sealing the slides, they were observed under a fluorescent microscope (Lecia DM-2500).

### The antitumor effects of scFv-OA2-si*RAPSYN* in subcutaneously transplanted tumor model

A mixture of 1*10^6 K562 cells at logarithmic growth stage with matrix gum (Beyotime, Shanghai, China) was injected into the lateral abdomen of 4-week-old NCG female mice. When the tumor volume reached ~ 50 mm^3^, the mice were randomly divided into 4 groups: saline, scFv-OA2-siNC, OA2-si*RAPSYN*, and scFv-OA2-si*RAPSYN*. Ten μg per mouse was injected into the tumor every two days, and tumor volume and mouse weight were recorded. The mice were euthanized when the tumor volume reached 1500 mm^3^. The formula used to calculate tumor volume was: tumor volume = (length × width^2^)/2. The tumor tissues were dissected, fixed with 4% paraformaldehyde, embedded in paraffin, sliced and stained with hematoxylin and eosin (H&E) or TUNEL or Ki67. The sections were photographed using a Lecia fluorescent microscope.

### Survival test in xenografted mouse model

To establish a xenografted mouse model, K562 cells were infected with lentivirus Fluc-GV260 (Genechem, Shanghai, China). K562 cells with stable expression of luciferase were screened out using Puromucin (Beyotime, Shanghai, China) to obtain Luc^+^ K562 cells. Then, 1 × 10^7 /200 μl K562 cells or Luc^+^ K562 cells were injected into the tail vein of 4-week-old NCG female mice. Forty mice with K562 cells were randomly divided into 4 groups (saline, scFv-OA2-siNC, OA2-si*RAPSYN*, and scFv-OA2-si*RAPSYN)*. Twelve mice with Luc^+^ K562cells were also randomly divided into corresponding 4 groups (n = 3). After 7 days, 2.5 nmol of different siRNA preparations were administered via caudal vein of the mice in each group. LNP-siRNA was administered every two days and the survival time of each mouse was recorded. The fluorescence of luc^+^K562 xenografted mice was monitored by IVIS Lumina III (PerkinElmer, MA, USA) in vivo imaging system at the same time. At the end of the experiments, before taking photography, potassium salt (Beyotime, Shanghai, China) was intraperitoneally injected to cause the death of all mice.

### Biodistribution of scFv-OA2-si*RAPSYN*

Nine mice with xenografted leukemia were randomly divided into three groups: siRNA, OA2-si*RAPSYN*, and scFv-OA2-si*RAPSYN*. Mice were treated with whole-body hair removal, and 2.5 nmol Cy5-siRNA was administered through tail vein in each group. Fluorescence intensity of the mice was detected by using IVIS® Spectrum Imaging System (PerkinElmer, MA, USA) at 0, 3, 6 and 24 h, and isoflurane was used for anesthesia. After 24 h of administration, heart, liver, spleen, lung, kidney and femoral head of the mice were collected, and the fluorescence values of Cy5-siRNA in each organ were detected by ex vivo imaging.

### Statistical analysis

All statistical analyses were performed using GraphPad Prism 8.0 (GraphPad Software Inc., CA, USA). Image J software was used for semi-quantitative analysis of protein bands. Data were compared using student T-test between two groups and ordinary univariate analysis of Variance (ANOVA) between three or more groups. The difference was considered statistically significant when *P < 0.05, **P < 0.01, ***P < 0.001, ****P < 0.0001 and “ns” indicated no significance (two-tailed distribution).

## Results

### Effective silencing of RAPSYN gene in Ph^+^ leukemia cell lines by OA2-si***RAPSYN***

To identifying the siRNA silencing effects in Ph^+^ leukemia cell lines, we first compared three siRNA sequences that target *RAPSYN* gene for their silencing efficiency (Additional file 1: Table S2). When individual siRNA was transfected into K562 cells with commercial liposomal transfection reagent Lipo2000 for 48 h, total RNA and proteins were extracted for qRT-PCR and Western blotting analyses. The results showed that sequence #3 exhibited the best silencing effects on RAPSYN expression (Additional file 1: Fig. S1).

To further improve the silencing effects of siRNA, three lipoid materials, including OA2 with a primary amine head group, TA7 and TA13 with a tertiary amine head group, were compared (Additional file 1: Fig. S2) [19]. These liposomal materials could load siRNA well without a leakage when N/P was above 5 (Additional file 1: Fig. S3). Then, same quantity of siRNA loaded with these liposomes and commercial liposome of RiboFECT™ CP (Ribo) was transfected to K562 cells to compare the transfection efficiency. After transfecting K562 cells by these lipoid-based lipid nanoparticles, OA2 showed a higher gene silencing efficiency and stronger inhibition of cell proliferation compared to TA7, TA13 and the commercial transfection reagent (Additional file 1: Fig. S4). In the Western blots, RAPSYN appeared two closely related bands, which is due possibly to the SRC-mediated phosphorylation [8]. Subsequently, to ascertain the transfection efficiency, these lipoid-based lipid nanoparticles were further evaluated in different cells. Flow cytometry analyses indicated that only OA2-based lipid nanoparticles could consistently deliver siRNA into all leukemia cell lines, including K562, MEG-01 and KU812 (Additional file 1: Fig. S5). Therefore, OA2-based lipid nanoparticles were chosen as the carrier for delivering si*RAPSYN* into Ph^+^ leukemia cell lines.

**Fig. 1:**
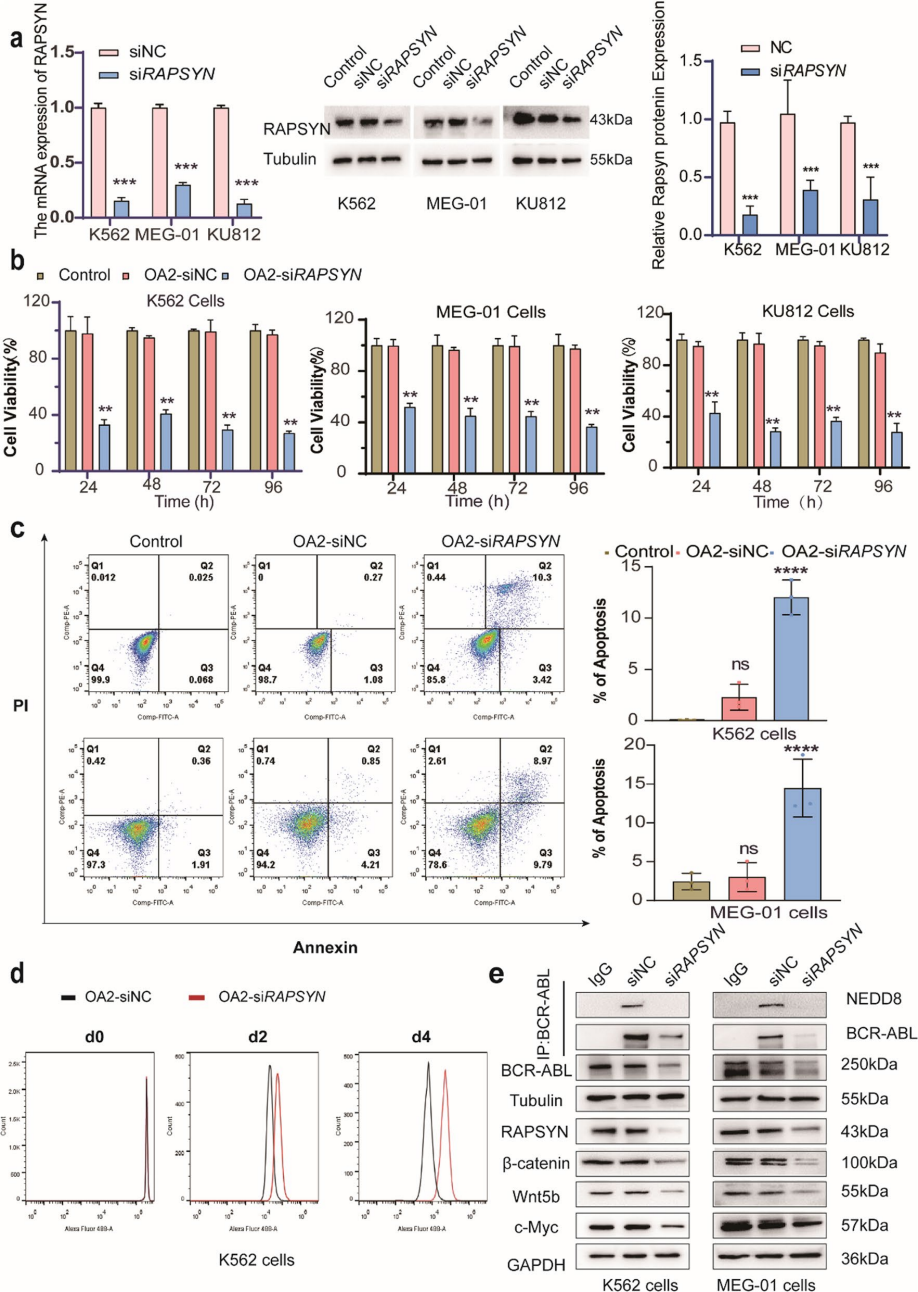
Inhibition of growth and proliferation of Ph^+^ leukemia cell lines by OA2-si*RAPSYN*. **a** The effects of OA2-si*RAPSYN* on mRNA level of *RAPSYN* gene and protein level of RAPSYN, after the Ph^+^ leukemia cell lines were treated with OA2-siNC or OA2-si*RAPSYN* for 24 h; **b** The effects of OA2-si*RAPSYN* on the viability of leukemia cell lines; **c** The effects of OA2-si*RAPSYN* on the apoptosis of leukemia cell lines; **d** The effects of OA2-si*RAPSYN* on the proliferation of K562 cells; **e** The effects of OA2-si*RAPSYN* on BCR-ABL neddylation and the expression of the proteins involved in Wnt/β-catenin/c-Myc signaling pathway. Statistical analyses were performed by student T-test. Data are presented as mean ± SD (n = 3). ns: no significance, **P < 0.01, ***P < 0.001, ****P < 0.0001 versus control

Next, the siRNA-based lipid nanoparticles (OA2-si*RAPSYN*) were prepared, showing an average particle size of 130.63 ± 2.48 nm and a positive charge of 25.46 ± 4.50 mV (Additional file 1: Table S3), as well as a similar morphology to blank OA2 lipid nanoparticles under transmission electron microscopy (Additional file 1: Fig. S6). Next, qRT-PCR and Western blotting were employed to examine the gene silencing effects on Ph^+^ leukemia cell lines. Indeed, OA2-si*RAPSYN* could efficiently deliver siRNA into all cells, resulting in approximately 80% of gene silencing efficiency (Fig. 1a). Meanwhile, the viability of Ph^+^ leukemia cell lines was obviously inhibited by the treatment of OA2-si*RAPSYN* (Fig. 1b), and the apoptosis of Ph^+^ leukemia cell lines was also increased after the treatment (Fig. 1c and Additional file 1: Fig. S7). In addition, we examined the proliferation of Ph^+^ leukemia cell lines on day 2 and day 4 after the treatment of OA2-siNC or OA2-si*RAPSYN*. The results showed that the proliferation ability of the cells was significantly inhibited by OA2-si*RAPSYN* (Fig. 1d and Additional file 1: Fig. S8). Together, silencing *RAPSYN* gene for the decrease of RAPSYN protein expression effectively led the inhibition of proliferation and the promotion of apoptosis for Ph^+^ leukemia cell lines.

### Inhibition of growth and proliferation of Ph^+^ leukemia cell lines by OA2-si***RAPSYN*** via Wnt/β-catenin/ c-Myc signaling pathway

To dissect the relationship between *RAPSYN* gene silencing and the suppression of cell viability of K562 cells, the neddylation associated BCR-ABL degradation and the Wnt/β-catenin/c-Myc signaling pathway primarily responsible for the cells proliferation and apoptosis were respectively examined. As shown in Fig. 1e, the silencing of *RAPSYN* gene resulted in the decrease of BCR-ABL neddylation and the increase of its degradation. Since Wnt/β-catenin/c-Myc signaling pathway is closely related to cell proliferation and apoptosis, the expression levels of these proteins were individually determined after silencing *RAPSYN* gene. Since the strong tyrosine kinase activity of BCR-ABL can phosphorylates Y654 of β-catenin to increase its stability and nuclear translocation [20], the translocation of β-catenin to the nucleus promotes the transcription of c-Myc gene to drive tumor progression [21]. Conversely, the decrease of BCR-ABL can lead to the reduction of β-catenin at cellular level, resulting in the inhibition of the expression of c-Myc to facilitate the process of cell apoptosis [22]. Thus, the present results suggested that the interference of *RAPSYN* gene transcription affects the viability of leukemia cell lines through the downregulation of Wnt/β-catenin/c-Myc signaling pathway.

### Expression and purification of anti-CD79B-scFv for targeting Ph^+^ leukemia cell lines

**Fig. 2:**
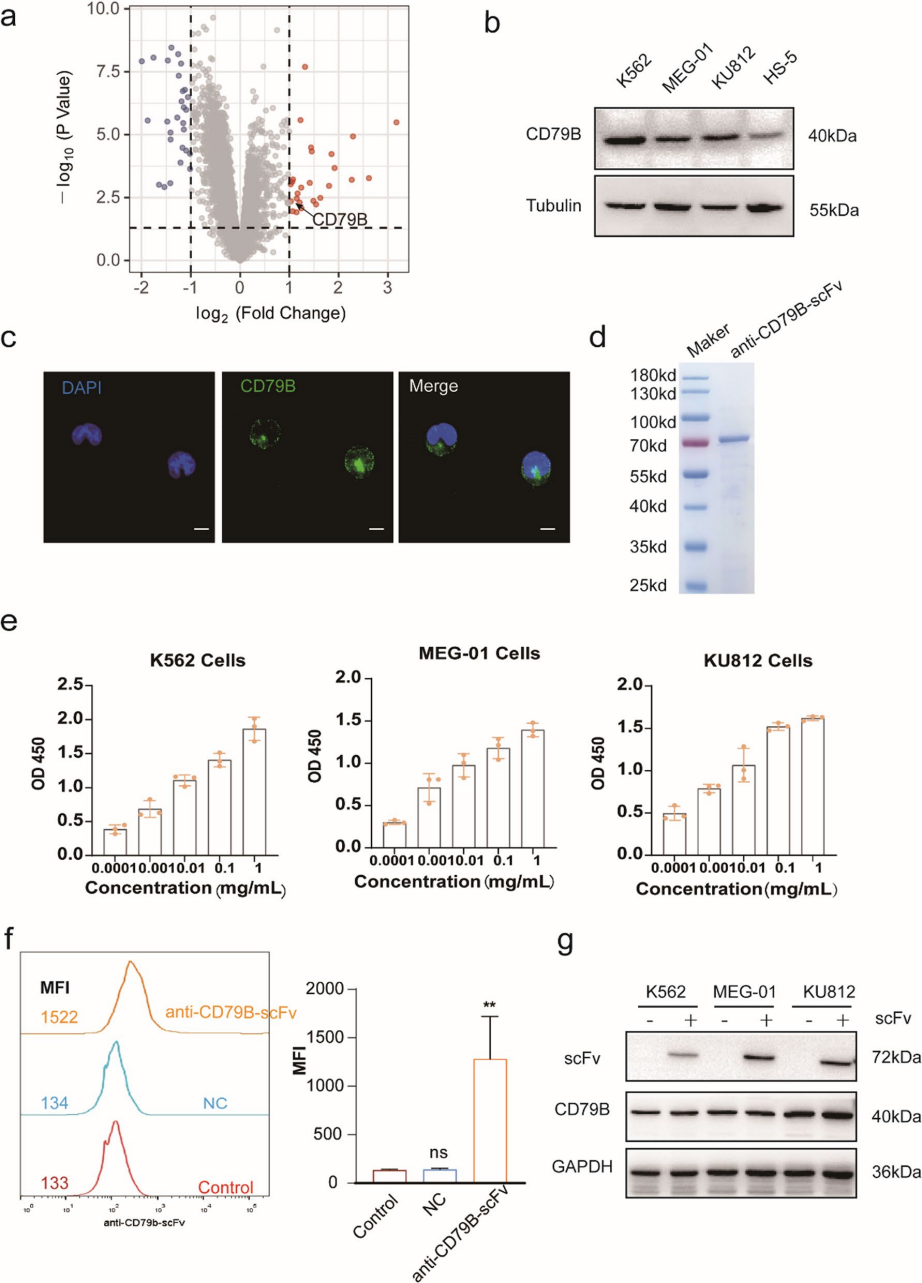
Expression, purification and binding affinity of anti-CD79B-scFv. **a** Volcano map of whole gene expression matrix (GSE555) for CML patients. GEO2R was used for online analysis of the differential gene datasets; **b** The expression level of CD79B in leukemia cell lines detected by Western blotting. **c** Localization of CD79B (green) in K562 cells (scale: 10 μm). **d** SDS-PAGE of purified anti-CD79B-scFv with Coomassie blue staining; **e** The binding capability of anti-CD79B-scFv with leukemia cell lines detected by ELISA; **f** The binding of anti-CD79B-scFv with K562 cells determined by flow cytometry; **g** Binding specificity of anti-CD79B-scFv with different cells detected by Western blotting. ns: no significance, **P < 0.01 versus control group

To identify a specific marker on the surface of Ph^+^ leukemia cells for possible targeted therapy, whole-genome expression matrix (GSE5550) was applied to compare the differences of CD34^+^ cells in the bone marrow of untreated chronic myeloid leukemia (CML) patients and healthy volunteers in GEO database. Differential gene enrichment and analysis revealed that *CD79B* gene is highly transcribed in CML patients (Fig. 2a). Subsequently, the expression of CD79B protein in Ph^+^ leukemia cell lines was confirmed by Western blotting, and higher expression levels of CD79B in Ph^+^ leukemia cell lines were further verified by the comparison with human bone marrow stromal cell line of HS-5 (Fig. 2b). More importantly, CD79B was localized on the surface of K562 cells by fluorescent microscopic analysis (Fig. 2c), suggesting that CD79B could be an ideal outer membrane protein for specific binding.

Due to small size and the versatility for different conjugations, single-chain fragment variable (scFv) region of anti-CD79B monoclonal antibody was chosen for the binding with CD79B on the surface of Ph^+^ leukemia cell lines. To express this scFv in *Escherichia coli*, MBP and 6xHis tags were respectively fused at N- and C-termini of the anti-CD79B-scFv sequence for soluble expression. After the induction by IPTG, anti-CD79B-scFv protein (72 kDa) was expressed in the soluble form. Then, two-step purification process, consisting of nickel affinity chromatography and dextrin-sepharose affinity chromatography, was employed to purify the protein, and the purity of anti-CD79B-scFv was greater than 95% judged by SDS-PAGE (Fig. 2d). To determine the binding capability of anti-CD79B-scFv with Ph^+^ leukemia cell lines, ELISA and FACS were used for the examination. ELISA results indicated that anti-CD79B-scFv binds to Ph^+^ leukemia cell lines in a concentration-dependent manner (Fig. 2e). After incubating anti-CD79B-scFv with K562 cells for 1 h, the fluorescent responses to anti-His fluorescent antibody of the cells were significantly shifted from the measurements by FACS (Fig. 2f). However, when HS-5, a normal human bone marrow stromal cell line with low expression of CD79B, was treated with anti-CD79B-scFv in the same way, and the results of FACS showed no statistical difference compared to NC (Additional file 1: Fig. S9). To exclude non-specific binding and false positives, total proteins of each type of cells with or without anti-CD79B-scFv were used for Western blotting. As shown in Fig. 2g, anti-CD79B-scFv could specifically bind with CD79B on the cell surface, demonstrating the utility of anti-CD79B-scFv as a valuable tool for targeted binding.

### Preparation and characterization of scFv-OA2-si*RAPSYN*

To exhibit CD79B targeting ability, the anti-CD79B-scFv should conjugate on the surface of OA2-si*RAPSYN* for targeting. For the conjugation, OA2 and DSPE-PEG2000-Maleimide (DPM) at a ratio of 98/2 (mol:mol) were mixed to obtain the lipid nanoparticles (OA2-DMP) by thin film extrusion. Then, the C-terminal cysteine in anti-CD79B-scFv was reacted with the maleimide group of OA2-DMP. Next, the ratio of anti-CD79B-scFv to OA2-DMP was optimized. When the ratio was about 0.06% (mol:mol), the resulting scFv-OA2 showed the most suitable particle size (Additional file 1: Table S4), and the best siRNA delivery efficiency (Additional file 1: Fig. S10). Notably, the coupling rate was determined to be 92.49% by BCA assay (Additional file 1: Fig. S11).

**Fig. 3:**
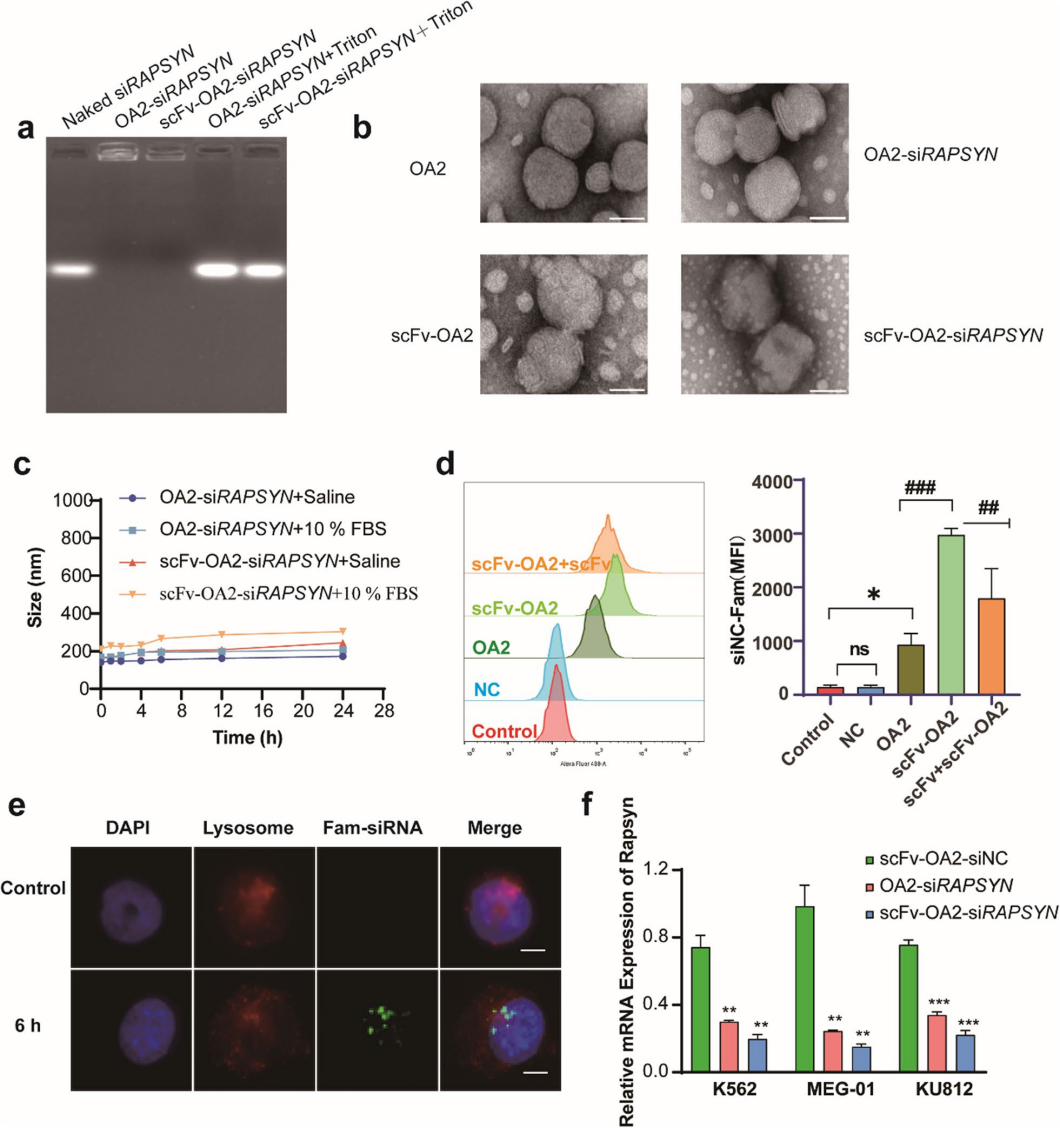
Preparation and characterization of anti-CD79B-scFv-OA2-si*RAPSYN*. **a** Agarose gel electrophoresis of OA2 and scFv-OA2 loaded with siRNA at N/P ratio of 5. **b** TEM images of OA2, OA2-si*RAPSYN*, scFv-OA2 and scFv-OA2-*siRAPSYN* (scale: 50 μm). **c** Stability of different lipid nanoparticles in saline and medium containing 10% (v/v) FBS. **d** Flow cytometry of cellular uptake of FAM-siRNA via different formulations by K562 cells. Statistical analysis was based on MFI values (n = 3). **e** The separation between lysosomes (red) and intracellular FAM-siRNA (green) in K562 cells under fluorescent microscope (scale: 5 μm). **f** Comparisons of mRNA levels of *RAPSYN* after the treatment of different formulations. Data are presented as mean ± SD (n = 3). ns: no significance, *P < 0.05, **P < 0.01 versus control; ^###^P < 0.001,^##^P < 0.01 versus scFv-OA2

Subsequently, scFv-OA2 was complexed with si*RAPSYN* to generate scFv-OA2-si*RAPSYN.* Agarose gel electrophoresis showed that, scFv-OA2 and OA2 could tightly bind si*RAPSYN* without leakage at a N/P ratio of 5 because no siRNA band was found in the groups of scFv-OA2-si*RAPSYN* and OA2-si*RAPSYN*, whereas siRNA bands obviously presented on the gels after Triton treatment to destroy the lipoplex (Fig. 3a). Meanwhile, TEM images showed that, scFv-OA2 and scFv-OA2-si*RAPSYN* were larger than that of OA2 and OA2-si*RAPSYN*, respectively, owing to the conjugation of anti-CD79B-scFv (Fig. 3b), which was similar to the observations from dynamic light scattering (Additional file 1: Table S5). In addition, when scFv-OA2-si*RAPSYN* was incubated in saline or 10% FBS medium for 24 h, their particle sizes did not show significant changes (Fig. 3c), indicating the formation of stable particles in vitro. Collectively, these physical characteristics made scFv-OA2-si*RAPSYN* possible for cellular uptake and gene silencing.

### Cellular uptakes of scFv-OA2-si*RAPSYN*

To test whether scFv-OA2-si*RAPSYN* could effectively enter Ph^+^ leukemia cell lines, the uptake of FAM-siRNA via different lipid nanoparticles by K562 cells were detected by flow cytometry. As shown in Fig. 3d, scFv-OA2 could deliver si*RAPSYN* into cells more efficiently than OA2 alone, and its MFI value was significantly higher than that from OA2. To confirm that this difference on MFI was related to anti-CD79B-scFv, free scFv was added to compete scFv-OA2-si*RAPSYN* in K562 cells. As a result, the significant decrease of MFI value indicated that excessive amount of scFv could hamper the cellular uptake of scFv-OA2-si*RAPSYN*, further demonstrating profound positive impacts of anti-CD79B-scFv conjugation on the uptake of scFv-OA2 by K562 cells.

On the other hand, siRNA works at the free form to perform the function of gene silencing after the endocytosis of lipid particles containing siRNA in the cells. In the case of scFv-OA2-si*RAPSYN*, it was required to quickly release the siRNA in cytosol for its gene silencing. To examine the dissociation of si*RAPSYN* from lysosomes, FAM-siRNA signal (Green) of scFv-OA2-si*RAPSYN* and LysoTracker signal (Red) in K562 cells were visualized to observe the locations under fluorescent microscopy. After incubating with K562 cells for 6 h, complete dismissal of the signals of Fam-siRNA and LysoTracker Red was observed, confirming the escape of scFv-OA2-siRNA from lysosomes (Fig. 3e). To evaluate whether scFv-OA2-siRNA can overcome the biological barriers encountered by siRNA to exert gene silencing effects, mRNA levels of *RAPSYN* in Ph^+^ leukemia cell lines were quantified by qRT-PCR after the treatment with scFv-OA2-si*RAPSYN* for 24 h. As shown in Fig. 3f, scFv-OA2-si*RAPSYN* could effectively decrease mRNA levels of *RAPSYN* in all three tested Ph^+^ leukemia cell lines, showing the benefit of anti-CD79B-scFv conjugation. These results provided evidence on the effectiveness of gene silencing by scFv-OA2-si*RAPSYN* at cellular levels.

### Anti-tumor effects of scFv-OA2-si*RAPSYN* in xenografted mice

**Fig. 4:**
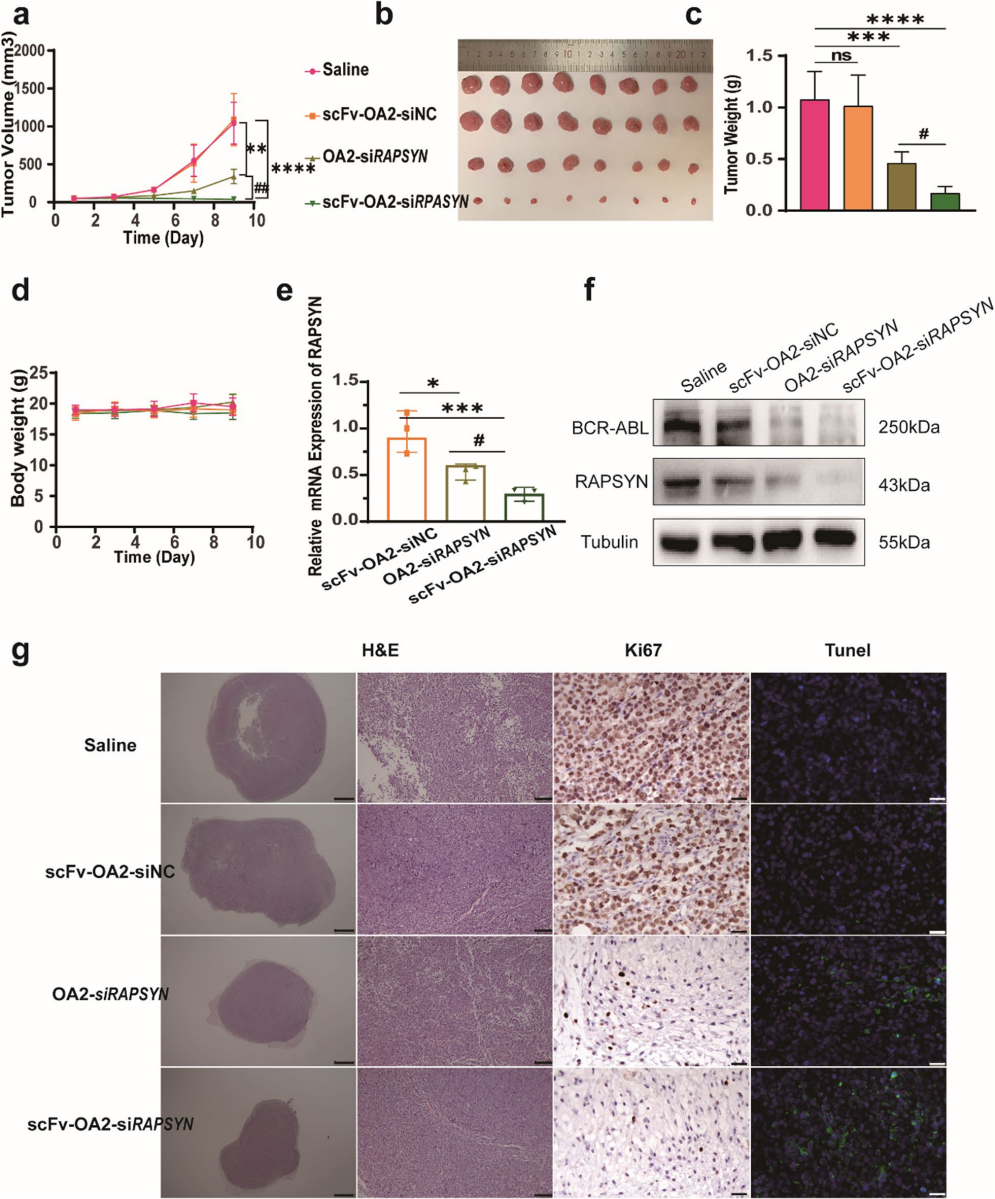
Inhibitory effects of scFv-OA2-si*RAPSYN* in xenografted mouse tumor model. **a** Changes of tumor volume during the treatments by time. **b** Photograph of tumor size after the treatments. **c** Statistical analysis of tumor weights after the treatments. **d** Changes in body weight from day 1 to day 9 after administration. **e** mRNA levels of *RAPSYN* in dissected tumor tissues by qRT-PCR. **f** Protein levels of RAPSYN and BCR-ABL in tumor tissues by Western blotting. **g** H&E staining (scale: left 1 mm; right 100 μm), Ki67 staining (scale: 25 μm) and Tunel staining (scale: 25 μm) of tumor tissues. Data are presented as mean ± SD (n = 8). ns: no significance, *P < 0.05, **P < 0.01, ***P < 0.001, ****P < 0.0001 versus control; ^#^P < 0.05 versus OA2-si*RAPSYN*

To assess if the cellular effects of scFv-OA2-si*RAPSYN* could translate to anti-tumor activity in vivo, a xenografted mouse model was established by subcutaneously implanting K562 cells. Once the tumor grew to approximately 50 mm^3^, scFv-OA2-si*RAPSYN* and different control groups were intratumorally administrated in the mice every two days. As shown in Fig. 4a, compared to saline and NC groups, the tumor volumes of OA2-si*RAPSYN* and scFv-OA2-si*RAPSYN* groups increased slowly, and scFv-OA2-si*RAPSYN* group showed a more remarkable effect on the inhibition of tumor growth. After sacrificing the mice, tumor tissues were collected, photographed and weighed. The tumor weight was statistically analyzed, and the results were consistent with the data of tumor volume (Fig. 4b, c). Notably, scFv-OA2-si*RAPSYN* treatments resulted in more potent anti-tumor effects than the siRNA without scFv region, further confirming the critical roles of anti-CD79B-scFv for specific targeting and subsequent gene silencing. Meanwhile, body weight of the mice did not have significant changes, and no adverse effects were noticeably observed during and after the treatments (Fig. 4d).

To ascertain that the anti-tumor efficacy of scFv-OA2-si*RAPSYN* was resulted from the decrease of BCR-ABL neddylation and the increase of BCR-ABL degradation by the silencing of *RAPSYN* gene, tumor tissues in different groups of the mice were collected for various analyses. Both OA2-si*RAPSYN* and scFv-OA2-si*RAPSYN* were able to significantly lower the levels of mRNA and protein of the target gene *RAPSYN* (Fig. 4e), thereby promoting the degradation of BCR-ABL (Fig. 4f). In particular, scFv-OA2-si*RAPSYN* exhibited the best capability on gene silencing. In addition, H&E staining showed that scFv-OA2-*siRAPSYN* could reduce tumor cell density and blurred boundaries. Ki67 staining and TUNEL analysis also confirmed the inhibitory effects on tumor growth by scFv-OA2-si*RAPSYN* (Fig. 4g). Therefore, the anti-tumor effects of scFv-OA2-si*RAPSYN* were definitively confirmed in the cell line-based mouse model, and the advantage of anti-CD79B-scFv conjugation was verified in vivo.

### Prolonged survival of leukemic mice by scFv-OA2-si*RAPSYN*

**Fig. 5:**
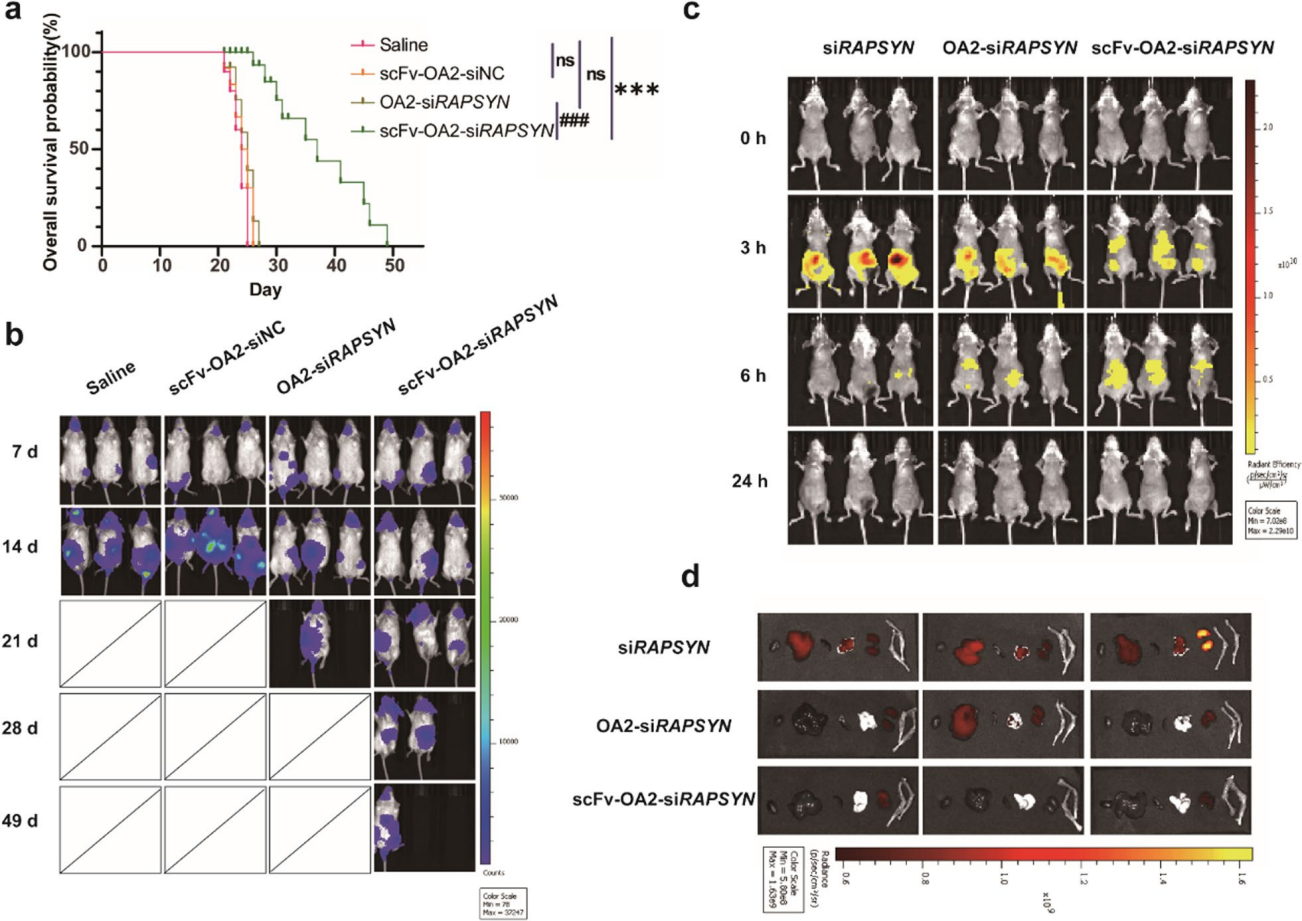
Therapeutic effects of scFv-OA2-si*RAPSYN* on human Ph^+^ leukemia mouse models of disease and distribution in vivo. **a** Kalpan-Meier survival curve of leukemic mice (n = 10), ns: no significance, ***P < 0.001 versus control group; ^##^P < 0.001 versus OA2-si*RAPSYN* group. **b** Fluorescence signals from K562 cells in mice with different treatments. **c** Biodistribution of Cy5-si*RAPSYN* over the time in vivo. **d** Distribution of Cy5-si*RAPSYN* in organs (from left to right: heart, liver, spleen, lung, kidney and femoral head) after the administrations for 24 h

To evaluate the therapeutic potential of scFv-OA2-si*RAPSYN* for targeted therapy of Ph^+^ leukemia, a Ph^+^ leukemic cell line-based mouse model was constructed by intravenously injecting K562 cells into the tail veins of NCG mice. After 7 days, the mice were intravenously administrated with different preparations every two days, and their survival times were recorded. OA2-si*RAPSYN* without scFv, similar to saline and scFv-OA2-siNC, showed no therapeutic effects. Compared to the controls, scFv-OA2-si*RAPSYN* could remarkably prolong the survivals of leukemic mice (Fig. 5a).

To directly monitor the disease progression of leukemic mice, K562 cells were constructed with fluorescence reporter gene to establish a fluorescent leukemic mouse model, and the malignant status and progression of the disease was regularly monitored. As shown in Fig. 5b, the imaging results were in accordance with the survival data. As time went by, the fluorescence signals of all control groups, including saline, OA2-si*RAPSYN* and scFv-OA2-siNC, increased sharply and gradually diminished after 20 days. By contrast, the florescence signals in scFv-OA2-si*RAPSYN* group were relative lower, indicating the inhibition of tumor growth. Collectively, these results again confirmed that OA2-si*RAPSYN* with anti-CD79B-scFv can specifically target leukemic cells to inhibit the progression of Ph^+^ leukemia.

### Biodistribution and safety of scFv-OA2-si*RAPSYN*

To investigate the distribution of scFv-OA2-si*RAPSYN* in leukemic mouse model, in vivo imaging was performed after administration of scFv-OA2-Cy5-si*RAPSYN* over the time. As shown in Fig. 5c, the fluorescence signals in all mice were mainly accumulated in the abdomen at 3 h after the administrations. At 6 h, the intensity of fluorescence signals in free si*RAPSYN* group was significantly weakened, whereas the groups of OA2-si*RAPSYN* and scFv-OA2-si*RAPSYN* still produced strong fluorescence, especially the scFv-OA2-si*RAPSYN* group. After 24 h, the fluorescence of Cy5-si*RAPSYN* was undetectable in all groups, indicating the complete clearance of siRNA within 24 h. With regard to the distribution of scFv-OA2-si*RAPSYN*, it was not resided in particular organs, which is likely due to its targeting effects in blood circulation. In parallel, the mice after administration were sacrificed at 24 h, and major organs were collected for ex vivo visualization of Cy5-si*RAPSYN* signal. As shown in Fig. 5d, strong fluorescence signals were found in liver, kidneys and lung in free siRNA group, whereas the fluorescence signals of OA2-si*RAPSYN* and scFv-OA2-si*RAPSYN* were largely located in the kidneys, implying that siRNA was liable to excrete through the kidneys.

To determine the practical utility of present approach, the safety of scFv-OA2-si*RAPSYN* was preliminarily evaluated in mice. Different si*RAPSYN* preparations were injected intravenously into leukemic mice every 2 days. After 10 days, the mice were sacrificed, and tissues were individually collected for pathological examinations. The examinations indicated that scFv-OA2-si*RAPSYN* exhibited good safety profiles in mice (Additional file 1: Fig. S13), which warrants further evaluations in different animal species for preclinical studies.

## Discussion

Despite the advancements and great successes of PROTAC-based design of small molecules or nucleic acid-based gene therapies, direct targeting Ph^+^ leukemia has not practically shown their therapeutic potentials [23–26]. Compared to the inhibition of tyrosine kinase activity by TKI, the degradation of oncoprotein BCR-ABL possesses obvious advantages to avoid drug resistance, relapse and recurrence. Previously, BCR-ABL was chosen as the first and the most important target for the demonstration of the feasibility of PROTAC technology by using various ubiquitin E3 ligases [27, 28]. Unfortunately, all excellent molecular and cellular activities on the degradation were unable to translate to hematological tumor models for prolonging the survival times of the animals. Our recent findings revealed that, in Ph^+^ leukemia cells, the Lys residues in BCR-ABL are modified by a scaffolding protein RAPSYN that possesses NEDD8 E3 ligase activity[8]. These neddylation modifications generates a shield on the surface of BCR-ABL oncoprotein to prevent the binding and degradation by PROTAC-based degraders, which provides a sensible explanation on the lack of in vivo efficacy of these degraders. Meanwhile, the discovery of RAPSYN-mediated neddylation of BCR-ABL also provides an alternative degradation approach because inhibition of the NEDD8 E3 ligase activity of RAPSYN or silencing of *RAPSYN* gene could achieve the reduction of BCR-ABL neddylation for the ease of c-CBL-mediated proteasomal degradation. However, specific inhibition of the NEDD8 E3 ligase activity of RAPSYN is an impossible task since RAPSYN protein is extensively distributed in various tissues [29]. Consequently, silencing of *RAPSYN* gene becomes an ultimate choice.

In the past 20 years, siRNA has emerged as a new therapeutic technology, which is regarded as a unique therapy for the treatment of broad-spectrum refractory diseases [30]. To accomplish desired therapeutic effects, efficient delivery of siRNA into cells is a prerequisite [31]. In this study, we chose OA2, a demonstrated effective cationic lipid nanoparticle with high transfection efficiency [32], as the carrier to deliver the siRNA against *RAPSYN* gene, which has shown excellent *RAPSYN* gene silencing in Ph^+^ leukemia cell lines. In addition, OA2-si*RAPSYN* could significantly inhibit the proliferation of Ph^+^ leukemia cell lines, through the regulation of Wnt/β-catenin/c-Myc signaling pathway, which is another advantage to possibly eliminate leukemia stem cells in Ph^+^ leukemia patients [33–35].

Given the nature of hematological malignancy, Ph^+^ leukemia cells continuously distribute in various issues. To effectively deliver sufficient amount of siRNA into cells, specific binding between siRNA delivery system and leukemia cells would be essential for achieving promising therapeutic effects. For example, because IL1 receptor-associated protein (IL1RAP) is a tumor-associated antigen on cell surface, targeting this antigen was attempted to eradicate leukemia stem cells, but several issues of toxicity and side effects were associated with its lack of specificity [36]. In our case, specifically targeting a surface marker on Ph^+^ cell lines could offer precise recognition for OA2-si*RAPSYN* by Ph^+^ leukemia cells, thus leading to effective *RAPSYN* silencing and minimized potential adverse effects. Indeed, the analysis of GEO databases indicated that CD79B protein could be a candidate of surface antigen based on its high expression on leukemic cell surface. In addition, the safety of anti-CD79B monoclonal antibody has been clinically demonstrated, as exemplified by marketed drugs [37, 38]. Therefore, CD79B was selected in this study as a surface molecule to specifically bind the scFv portion of its antibody, which should be able to result in targeted therapy for Ph^+^ leukemia. Indeed, OA2-si*RAPSYN* with anti-CD79B-scFv as the ligand showed targeting capability with specific uptakes by Ph^+^ leukemia cell lines for *RAPSYN* gene silencing and BCR-ABL degradation, conforming the druggable value of targeting *RAPSYN* gene. The effectiveness of scFv-OA2-si*RAPSYN* was also verified by tissue distribution, anti-tumor efficacy and survival time in leukemic cell line-based mouse models. Compared to other approaches of gene therapy for hematological diseases, the conjugation of a scFv portion with lipid nanoparticles for specific targeting of a certain group of cells in the circulation is a remarkable advancement in the field, which could promote the design of novel therapeutic strategies and the development of more precise and sophisticated treatment options.

## Conclusions

In summary, we have designed and evaluated a dual-targeted scFv-OA2-si*RAPSYN* for gene therapy of Ph^+^ leukemia. Specific binding with Ph^+^ cell lines was accomplished by the conjugation of anti-CD79B-scFv with OA2-based lipid nanoparticles, thereby exhibiting effective gene silencing of *RAPSYN* for the degradation of oncogenic BCR-ABL. Moreover, the therapeutic values of scFv-OA2-si*RAPSYN* were demonstrated in xenografted mouse models. The present leukemic cell line-based study has not only offered a new protein degradation-based therapeutic strategy for the treatments of Ph^+^ leukemia, but also shown the potentials of expanding its utility for other hematological diseases.

### Supplementary Information

**Additional file 1: Figure S1.** The mRNA and protein expression levels of RAPSYN by si*RAPSYN* treatments. After transfected with different siRNA for 48 h, mRNA and protein levels were detected by qRT-PCR and Western blotting. Data are presented as mean ± SD (n = 3). ***P<0.001versus control group. **Figure S2.** Chemical structures of three lipoids. **Figure S3.** Agarose gel electrophoresis of liposome-siRNA. The red box indicates the lanes for intactness. **Figure S4.** Comparison of the expression of RAPSYN in K562 cells by different lipid carriers. The mRNA and protein levels of RAPSYN were detected by qRT-PCR and Western blotting after the transfection of different lipoid-based lipoplexes for 48 h. RiboFECT™ CP (Ribo) was used as the transfection reagent, and control indicates no treatment. Data are presented as mean ± SD (n = 3). *P<0.05 versus Ribo group. **Figure S5. **Cellular uptake of FAM-siRNA via different lipoid-based lipoplexes by K562 cells, MEG-01 cells and KU812 cells for 12 h detected by FACS. **Figure S6. **Transmission electron microscope images of OA2 and OA2-si*RAPSYN* (scale: 50 μm). **Figure S7.** Cell apoptosis of KU812 cells after treating with OA2-siNC or OA2-siRNA for 24 h examined by flow cytometry. Data are presented as mean ± SD (n=3). ns: no significance, ****P<0.0001 versus control group. **Figure S8.** Cell proliferation of MEG-01 and KU812 cells after treating with OA2-siNC or OA2-si*RAPSYN* for 24 h examined by flow cytometry. **Figure S9. **The binding of anti-CD79B-scFv with HS-5 cells determined by flow cytometry. Treatment with PBS is represented as Control. Data are presented as mean ± SD (n=3). ns: no significant difference. **Figure S10. **The mean fluorescence intensity (MFI) of FAM-siRNA via scFv-OA2 with different scFv ratios in K562 cells examined by flow cytometry. **Figure S11.** The coupling rate of anti-CD79B-scFv on the lipid nanoparticles. **Figure S12. **Representative images of H&E staining of the organs (heart, liver, spleen, lung and kidney) of each group (scale bars: 100 μm). **Table S1.** Codon optimized nucleic acid sequence of anti-CD79B-scFv. MBP portion is marked in red; scFv portion is in black; 6×His portion is colored in blue. **Table S2.** Sequences of siRNA targeting *RAPSYN*. **Table S3.** Characterization of OA2 and OA2-si*RAPSYN* (n = 3). **Table S4.** Characterization of scFv-OA2 with different scFv molar ratios (n = 3). **Table S5.** Characterization of scFv-OA2 and scFv-OA2-si*RAPSYN* (n = 3).

## Data Availability

No datasets were generated or analysed during the current study.
