# Supplementary material for: Targeted degradation of oncogenic BCR-ABL by silencing the gene of NEDD8 E3 ligase RAPSYN
Source: J Nanobiotechnology. 2024 May 13;22:247. doi: 10.1186/s12951-024-02505-5 (PMC11089668; doi:10.1186/s12951-024-02505-5)
Supplement: Supplementary file 1 — Additional file 1: Figure S1. The mRNA and protein expression levels of RAPSYN by siRAPSYN treatments. After transfected with different siRNA for 48 h, mRNA and protein levels were detected by qRT-PCR and Western blotting. Data are presented as mean ± SD (n = 3). ***P<0.001versus control group. Figure S2. Chemical structures of three lipoids. Figure S3. Agarose gel electrophoresis of liposome-siRNA. The red box indicates the lanes for intactness. Figure S4. Comparison of the expression of RAPSYN in K562 cells by different lipid carriers. The mRNA and protein levels of RAPSYN were detected by qRT-PCR and Western blotting after the transfection of different lipoid-based lipoplexes for 48 h. RiboFECT™ CP (Ribo) was used as the transfection reagent, and control indicates no treatment. Data are presented as mean ± SD (n = 3). *P<0.05 versus Ribo group. Figure S5. Cellular uptake of FAM-siRNA via different lipoid-based lipoplexes by K562 cells, MEG-01 cells and KU812 cells for 12 h detected by FACS. Figure S6. Transmission electron microscope images of OA2 and OA2-siRAPSYN (scale: 50 μm). Figure S7. Cell apoptosis of KU812 cells after treating with OA2-siNC or OA2-siRNA for 24 h examined by flow cytometry. Data are presented as mean ± SD (n=3). ns: no significance, ****P<0.0001 versus control group. Figure S8. Cell proliferation of MEG-01 and KU812 cells after treating with OA2-siNC or OA2-siRAPSYN for 24 h examined by flow cytometry. Figure S9. The binding of anti-CD79B-scFv with HS-5 cells determined by flow cytometry. Treatment with PBS is represented as Control. Data are presented as mean ± SD (n=3). ns: no significant difference. Figure S10. The mean fluorescence intensity (MFI) of FAM-siRNA via scFv-OA2 with different scFv ratios in K562 cells examined by flow cytometry. Figure S11. The coupling rate of anti-CD79B-scFv on the lipid nanoparticles. Figure S12. Representative images of H&E staining of the organs (heart, liver, spleen, lung and kidney) of each group (sc [file 12951_2024_2505_MOESM1_ESM.docx]

**Additional file 1**

**Targeted Degradation of Oncogenic BCR-ABL by**

**Silencing the Gene of NEDD8 E3 Ligase RAPSYN**

Yanzi Sun^a^, Yishu Wang^b^, Chunyan Liu^a^, Yingshuang Huang^b^,

Qiulin Long^b^, Caoyun Ju^b^*, Can Zhang^b^*, Yijun Chen^a^*

^a^ State Key Laboratory of Natural Medicines and Laboratory of Chemical Biology, China Pharmaceutical University, 639 Longmian Ave, Nanjing, Jiangsu Province, 211198, China

^b^ State Key Laboratory of Natural Medicines, Jiangsu Key Laboratory of Drug Discovery for Metabolic Diseases, Center of Advanced Pharmaceuticals and Biomaterials, China Pharmaceutical University, Nanjing 210009, P.R. China

*Corresponding authors

E-mail addres: [yjchen@cpu.edu.cn](mailto:yjchen@cpu.edu.cn) ,[zhangcan@cpu.edu.cn](mailto:zhangcan@cpu.edu.cn), [jucaoyun@cpu.edu.cn](mailto:jucaoyun@cpu.edu.cn)


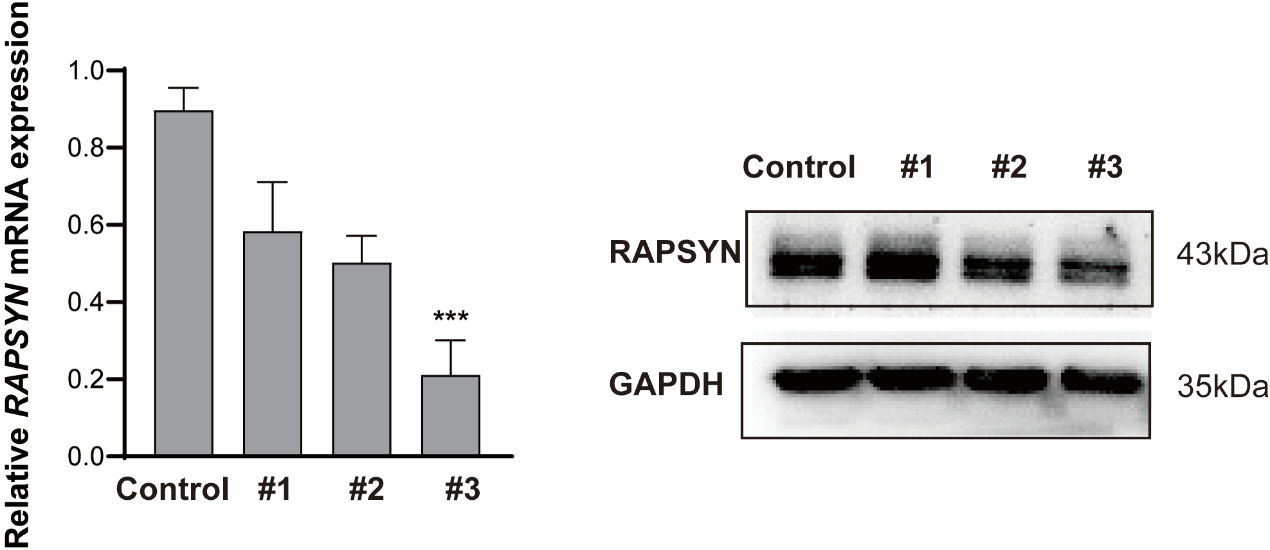


**Figure S1.** The mRNA and protein expression levels of RAPSYN by si*RAPSYN* treatments. After transfected with different siRNA for 48 h, mRNA and protein levels were detected by qRT-PCR and Western blotting. Data are presented as mean ± SD (n = 3). ***P<0.001versus control group.

**
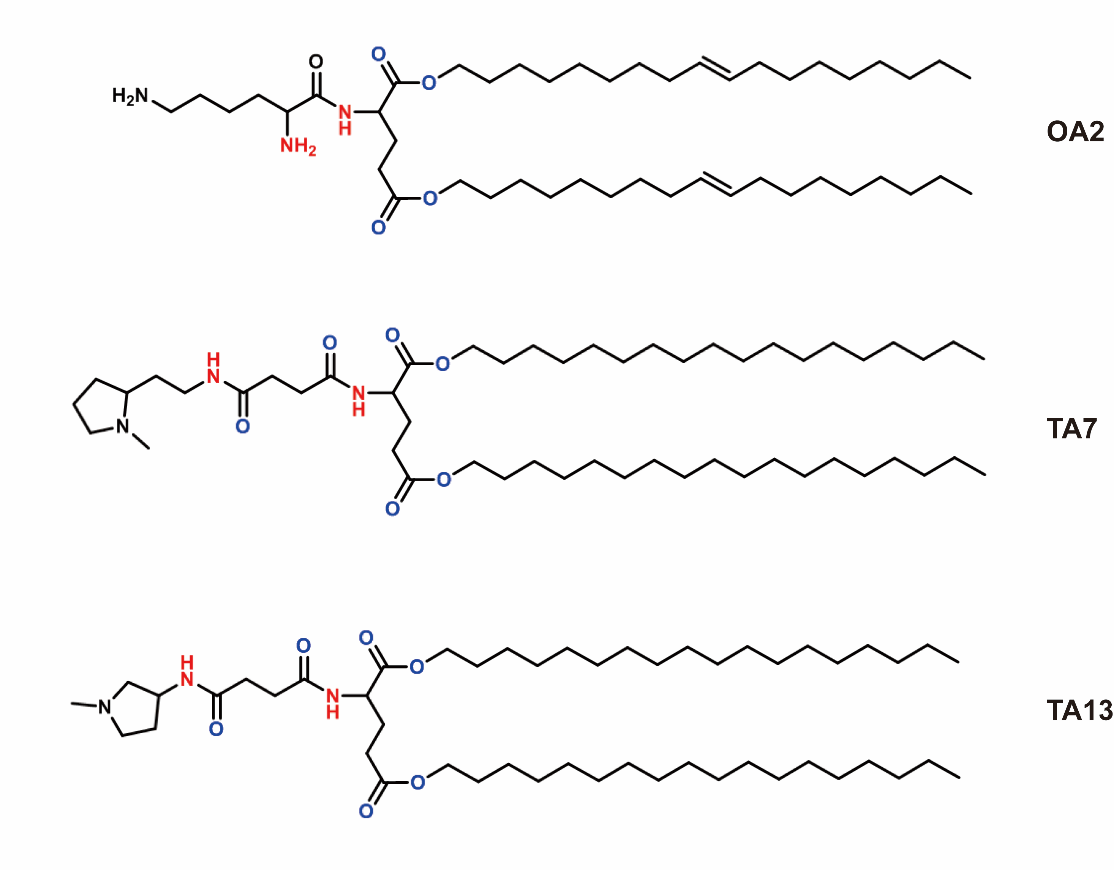
**

**Figure S2.** Chemical structures of three lipoids.


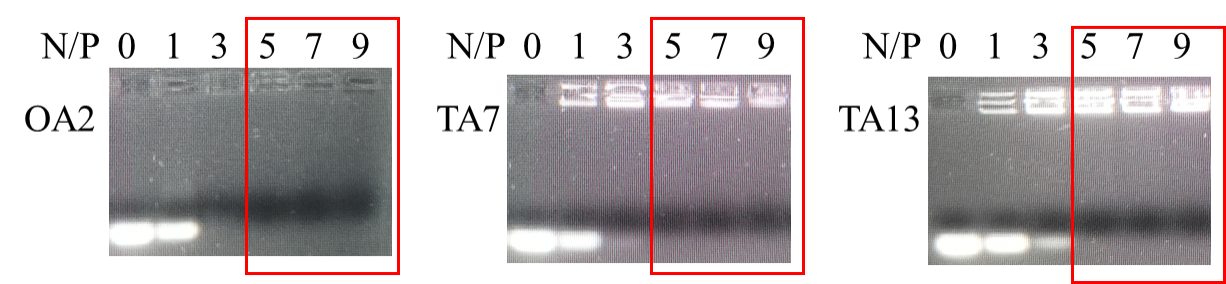


**Figure S3**. Agarose gel electrophoresis of liposome-siRNA. The red box indicates the lanes for intactness.


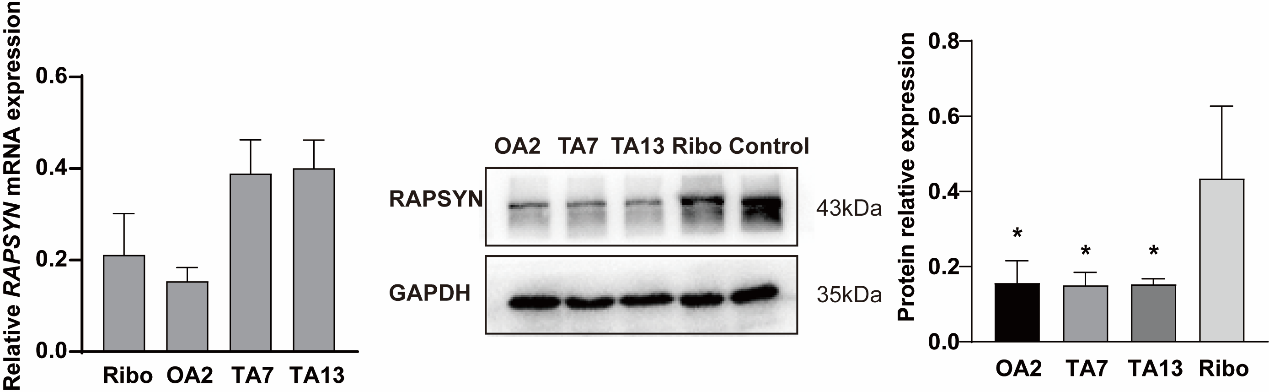


**Figure S4.** Comparison of the expression of RAPSYN in K562 cells by different lipid carriers. The mRNA and protein levels of RAPSYN were detected by qRT-PCR and Western blotting after the transfection of different lipoid-based lipoplexes for 48 h. RiboFECT™ CP (Ribo) was used as the transfection reagent, and control indicates no treatment. Data are presented as mean ± SD (n = 3). *P<0.05 versus Ribo group.


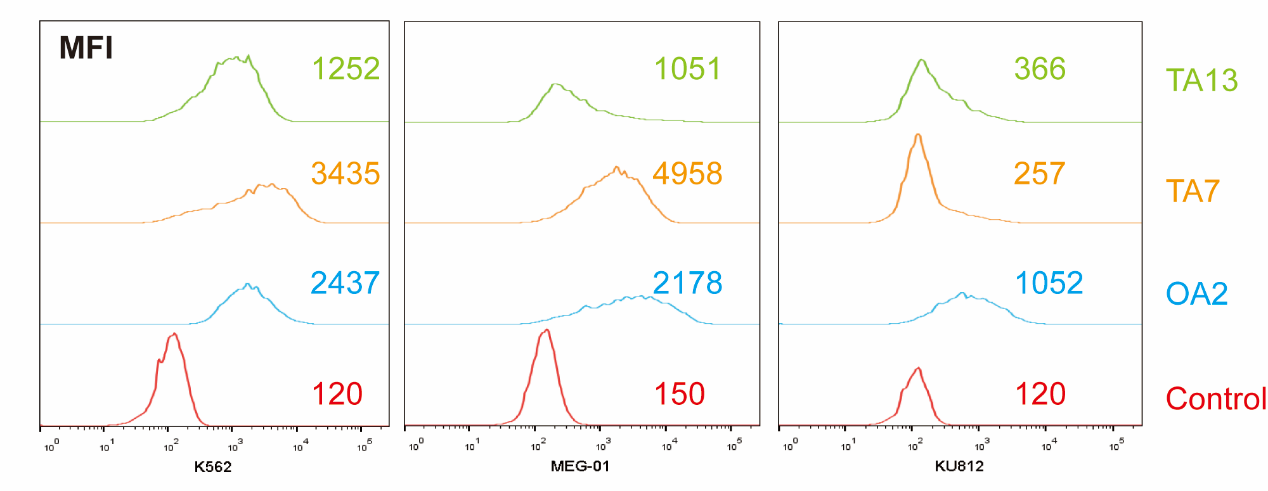


**Figure S5.** Cellular uptake of FAM-siRNA *via* different lipoid-based lipoplexes by K562 cells, MEG-01 cells and KU812 cells for 12 h detected by FACS.


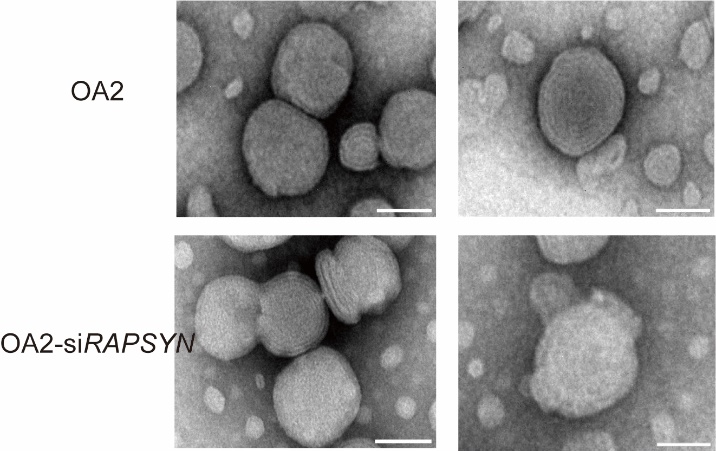


**Figure S6.** Transmission electron microscope images of OA2 and OA2-si*RAPSYN* (scale: 50 μm).


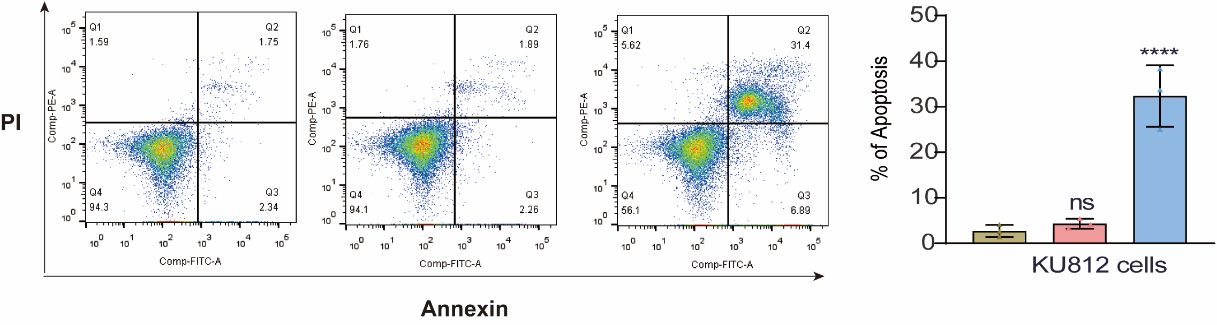


**Figure S7.** Cell apoptosis of KU812 cells after treating with OA2-siNC or OA2-siRNA for 24 h examined by flow cytometry. Data are presented as mean ± SD (n=3). ns: no significance, ****P<0.0001 versus control group.


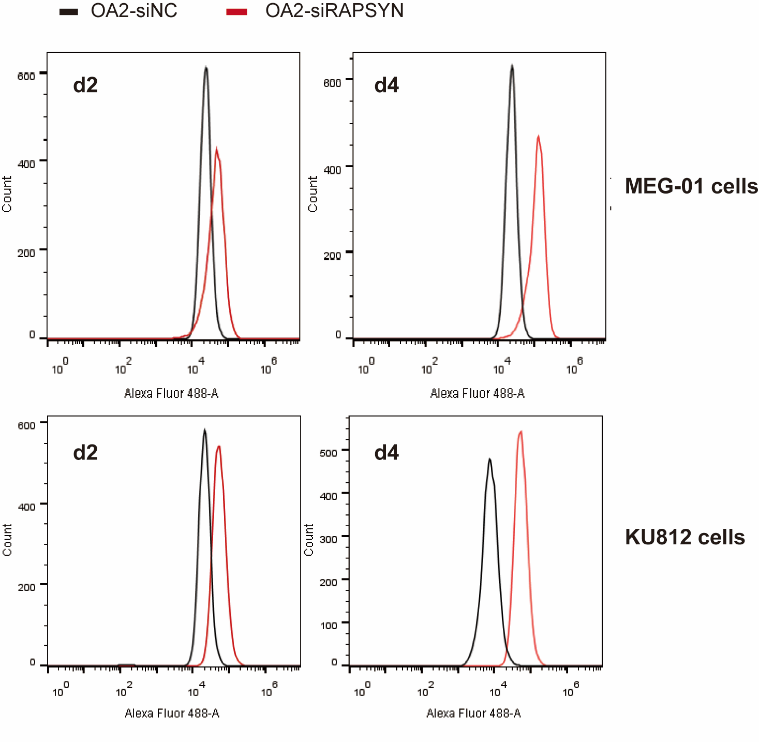


**Figure S8.** Cell proliferation of MEG-01 and KU812 cells after treating with OA2-siNC or OA2-si*RAPSYN* for 24 h examined by flow cytometry.


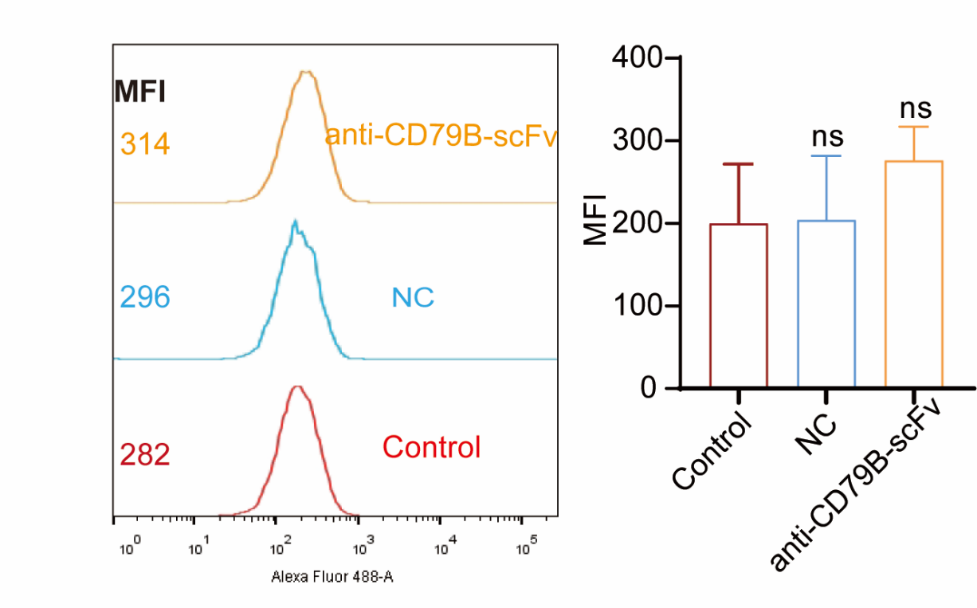


**Figure S9.** The binding of anti-CD79B-scFv with HS-5 cells determined by flow cytometry. Treatment with PBS is represented as Control. Data are presented as mean ± SD (n=3). ns: no significant difference.


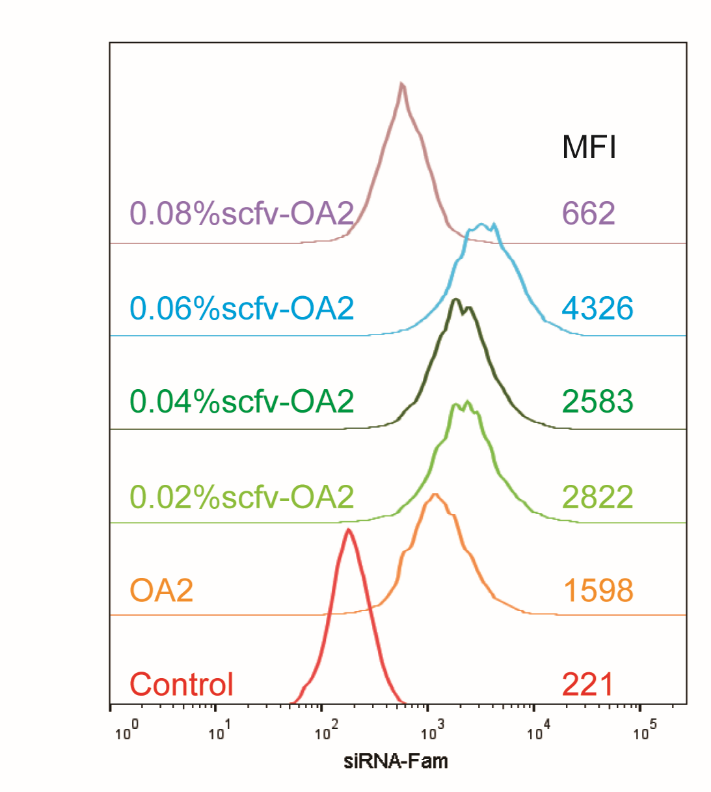


**Figure S10.** The mean fluorescence intensity (MFI) of FAM-siRNA *via* scFv-OA2 with different scFv ratios in K562 cells examined by flow cytometry.

OD_562_ (free anti-CD79B-scFv after the conjugation and centrifugation): 0.18

Coupling rate: 92.45% (conjugated anti-CD79B-scFv vs. total anti-CD79B-scFv)

**Figure S11.** The coupling rate of anti-CD79B-scFv on the lipid nanoparticles


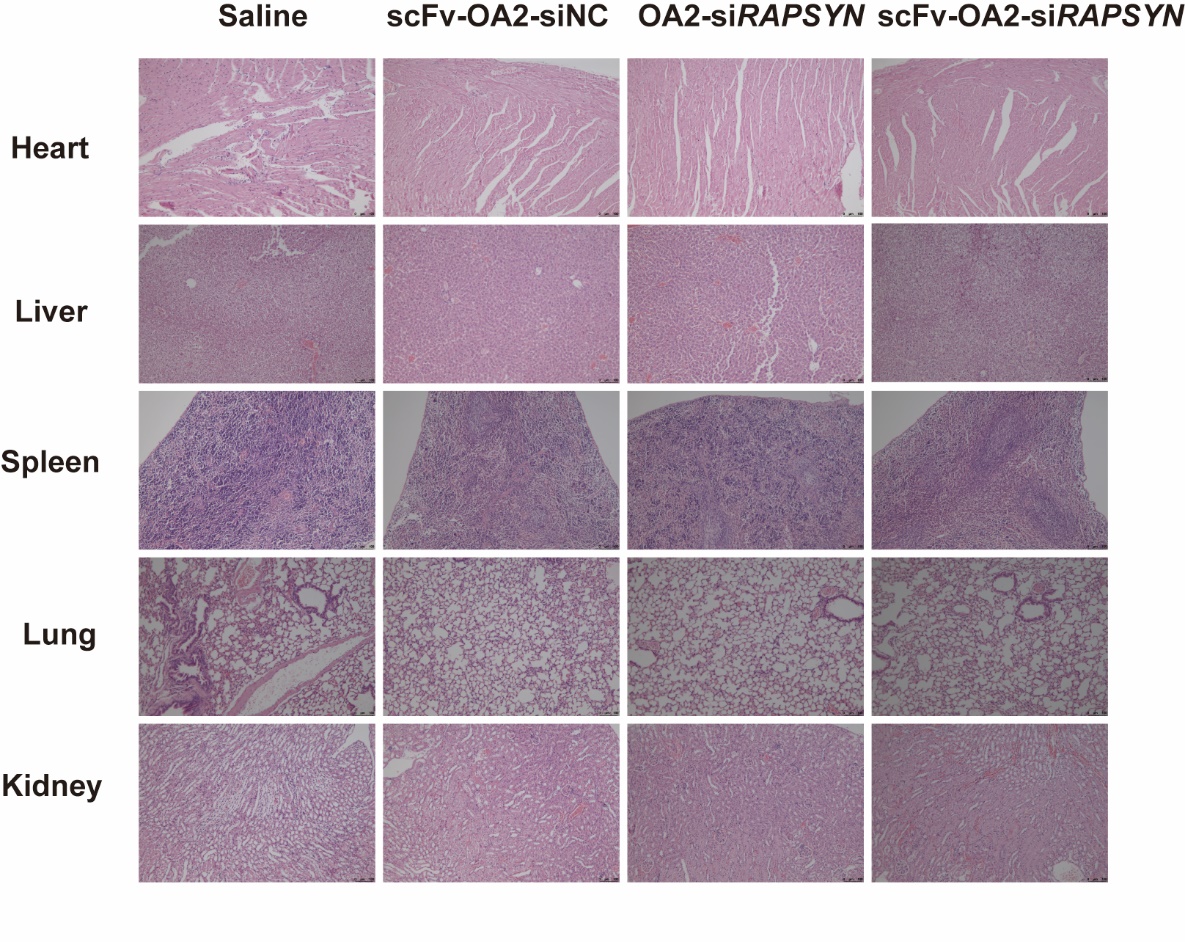


**Figure S12.** Representative images of H&E staining of the organs (heart, liver, spleen, lung and kidney) of each group (scale bars: 100 μm).

**Table S1.** Codon optimized nucleic acid sequence of anti-CD79B-scFv. MBP portion is marked in red; scFv portion is in black; 6×His portion is colored in blue.

aaaatcgaagaaggtaaactggtaatctggattaacggcgataaaggctataacggtctcgctgaagtcggtaagaaattcgagaaagataccggaattaaagtcaccgttgagcatccggataaactggaagagaaattcccacaggttgcggcaactggcgatggccctgacattatcttctgggcacacgaccgctttggtggctacgctcaatctggcctgttggctgaaatcaccccggacaaagcgttccaggacaagctgtatccgtttacctgggatgccgtacgttacaacggcaagctgattgcttacccgatcgctgttgaagcgttatcgctgatttataacaaagatctgctgccgaacccgccaaaaacctgggaagagatcccggcgctggataaagaactgaaagcgaaaggtaagagcgcgctgatgttcaacctgcaagaaccgtacttcacctggccgctgattgctgctgacgggggttatgcgttcaagtatgaaaacggcaagtacgacattaaagacgtgggcgtggataacgctggcgcgaaagcgggtctgaccttcctggttgacctgattaaaaacaaacacatgaatgcagacaccgattactccatcgcagaagctgcctttaataaaggcgaaacagcgatgaccatcaacggcccgtgggcatggtccaacatcgacaccagcaaagtgaattatggtgtaacggtactgccgaccttcaagggtcaaccatccaaaccgttcgttggcgtgctgagcgcaggtattaacgccgccagtccgaacaaagagctggcaaaagagttcctcgaaaactatctgctgactgatgaaggtctggaagcggttaataaagacaaaccgctgggtgccgtagcgctgaagtcttacgaggaagagttggcgaaagatccacgtattgccgccaccatggaaaacgcccagaaaggtgaaatcatgccgaacatcccgcagatgtccgctttctggtatgccgtgcgtactgcggtgatcaacgccgccagcggtcgtcagactgtcgatgaagccctgaaagacgcgcagactAATTCGAGCTCGAACAACAACAACAATAACAATAACAACAACCTCGGGATCGAGGAAAACCTGTATTTTCAGGGATCCCATATGGATATCCAGCTGACCCAGAGCCCGAGCAGCCTGAGCGCGAGCGTTGGTGATCGTGTTACCATCACCTGCAAAGCGAGCCAGTCTGTTGATTACGAAGGTGATAGCTTCCTGAACTGGTACCAGCAGAAACCGGGTAAAGCGCCGAAACTGCTGATCTACGCGGCGAGCAACCTGGAAAGCGGCGTTCCGTCCCGTTTCTCTGGCAGCGGTTCTGGTACCGATTTCACCCTGACCATCAGCAGCATCCAGCCGGAAGATTTCGCTACCTACTACTGCCAGCAGTCTAACGAAGATCCGCTGACCTTCGGCCAGGGCACCAAAGTTGAAATCAAACGTGGCGGCGGTGGTAGCGGCGGCGGCGGCTCCGGTGGCGGCGGCAGCGGCGGTGGTGGTTCTGAAGTTCAGCTGGTTGAATCTGGTGGCGGCCTGGTTCAGCCGGGTGGTAGCCTGCGTCTGAGCTGCGCGGCGTCTGGTTACACCTTCAGCAGCTACTGGATCGAATGGGTTCGTCAGGCGCCGGGCAAAGGCCTGGAATGGATCGGTGAAATCCTGCCGGGTGGTGGTGATACCAACTACAACGAAATCTTCAAAGGCCGTGCGACCTTCAGCGCGGATACCAGCAAAAACACCGCGTACCTGCAGATGAACAGCCTGCGTGCGGAAGATACCGCGGTTTACTACTGCACCCGTCGTGTTCCGATCCGTCTGGATTACTGGGGTCAGGGTACCCTGGTTACCGTTAGCTCTCTCGAGCACCACCACCACCACCAC

**Table S2.** Sequences of siRNA targeting *RAPSYN*

| Number | Sense（5'-3'） | Antisense（5'-3'） |
| --- | --- | --- |
|  |  |  |
| 1 | CATGAAGCCTGGCTTTGTA | UACAAAGCCAGGCUUCAUG |
| 2 | CGAGAAGCTGTGCGAGTTT | AAACUCGCACAGCUUCUCG |
| 3 | GCGCTATGCCCACAACAAT | AUUGUUGUGGGCAUAGCGC |

**Table S3.** Characterization of OA2 and OA2-si*RAPSYN* (n = 3)

| Characterization | | Particle size（nm） | Zeta potential（mV） | PDI |
| --- | --- | --- | --- | --- |
| OA2 | 136.12 ± 2.08 | | 42.79 ± 5.61 | 0.153 ± 0.023 |
| OA2-si*RAPSYN* | 130.63± 2.48 | | 25.46 ± 4.50 | 0.111 ± 0.033 |

**Table S4.** Characterization of scFv-OA2 with different scFv molar ratios (n = 3)

| scFv molar ratio (%) | | Particle size（nm） | Zeta potential（mV） | PDI |
| --- | --- | --- | --- | --- |
| 0.08 | 403.94 ± 14.10 | | -12.22 ± 1.64 | 0.220 ± 0.123 |
| 0.06 | 154.50 ± 1.40 | | 39.88 ± 3.96 | 0.193 ± 0.044 |
| 0.01 | 198.82 ± 5.38 | | 21.83 ± 6.33 | 0.280 ± 0.015 |
| 0 | 136.12 ± 2.08 | | 42.79 ± 5.61 | 0.153 ± 0.023 |

**Table S5.** Characterization of scFv-OA2 and scFv-OA2-si*RAPSYN* (n = 3)

| Characterization | Particle size（nm） | Zeta potential（mV） | PDI |
| --- | --- | --- | --- |
| scFv-OA2 | 154.50± 1.40 | 39.88 ± 3.96 | 0.193 ± 0.044 |
| scFv-OA2-si*RAPSYN* | 160.01± 3.43 | 28.87 ± 1.69 | 0.159 ± 0.061 |
